# Immediate impacts of COVID‐19 on female and male farmers in central Myanmar: Phone‐based household survey evidence

**DOI:** 10.1111/agec.12632

**Published:** 2021-05-03

**Authors:** Catherine Ragasa, Isabel Lambrecht, Kristi Mahrt, Zin Wai Aung, Michael Wang

**Affiliations:** ^1^ International Food Policy Research Institute (IFPRI) Washington DC USA; ^2^ International Food Policy Research Institute (IFPRI) Yangon Myanmar; ^3^ International Food Policy Research Institute (IFPRI) Colorado USA

**Keywords:** COVID‐19 impacts, gender, phone survey, rural livelihoods, women's empowerment

## Abstract

This article provides evidence of the immediate impacts of the first months of the COVID‐19 crisis on farming communities in central Myanmar using baseline data from January 2020 and follow‐up phone survey data from June 2020 with 1,072 women and men. Heterogeneous effects among households are observed. Fifty‐one percent of the sample households experienced income loss from various livelihood activities, and landless households were more severely affected by the crisis, mainly because of lost farm and nonfarm employment and negative impacts on rural enterprises. Women and men in these landless households were equally engaged and affected by lower wages or more difficulties in finding farm work; fewer women were engaged in nonfarm work, but almost all of them lost such nonfarm wage employment. Women in landless households are also particularly vulnerable in terms of worsened workload and increased tension in the household during COVID‐19. Landed households were also affected through lower prices, lower demand for crops, and difficulties in input access. Women and men differ in levels of stress, fear, and pessimism regarding the effects of COVID‐19. In most households, there were no signs that household task‐sharing and work balance improved, and no clear shift in intrahousehold relations was observed.

## 1 INTRODUCTION

Rural households and their members exhibit significant heterogeneity—across communities, within communities, and even within households. Access to rural infrastructure, landholdings and other assets, and income sources are often unequal (Davis et al., 2010), as are rural incomes, welfare, and well‐being (Reardon et al., 2000). Worldwide, major gender gaps have been reported in livelihood options, decision‐making power, and well‐being in rural households and communities (Quisumbing et al., 2014). Furthermore, a shock or crisis can affect such existing inequalities, for better or worse. Thus, an early understanding of how these inequalities might shift is imperative to a successful response to the current COVID‐19 crisis.

Rigorous evidence of the pandemic's impact is still scarce, but economic modeling forecasts that the pandemic will negatively affect global incomes and food security (Laborde et al., 2020). Studies on the immediate impact of COVID‐19 in Ethiopia and India have shown decreased consumption of more expensive foods, including more nutritious foods such as meat, fish, dairy, and vegetables (Harris et al., 2020; Hirvonen et al., 2020).

Many have warned that COVID‐19 will exacerbate existing inequalities in poverty, food and nutrition security, and well‐being (e.g., Adams‐Prassl et al., 2020; Laborde et al., 2020; Van Lancker & Parolin, 2020). In recent disease outbreaks such as Ebola, Zika, and SARS, responses did not consider gender and thus reinforced and aggravated existing gender inequalities. In particular, West Africa's Ebola virus outbreak in 2014–16 contributed to a dramatic increase in maternal mortality as Ebola responses diverted resources from reproductive and sexual health services toward emergency response (Sochas et al., 2017). Additionally, an assessment of the linkages between pandemics and violence against women and children shows that a large increase in this type of violence is expected during pandemics (Peterman et al., 2020).

This study reports on and analyzes the observed changes brought about by COVID‐19 and related response measures on the behavior and activities of women and men in Myanmar's Central Dry Zone between February and May 2020. Rural Myanmar, like elsewhere in South and Southeast Asia, has a significant population of landless households but also displays a diversity of livelihoods (Boutry et al., 2017). In the Dry Zone in particular, access to water—critical for crop production—is uneven (Boutry et al., 2017). Our study area includes nonfarm (landless) and farm (landed) households with and without water access for agriculture. For many households, both women and men supplement their income through engagement in farm and nonfarm wage employment and enterprises. Thus, disruptions in food systems, nonfarm employment, enterprises, migration, and remittances will likely affect the landless and landed, irrigation and nonirrigation households, and the livelihoods and welfare of women and men in multiple ways. Understanding the heterogeneous effects of the initial months of the COVID‐19 crisis on women's and men's activities and welfare will help policy makers and practitioners design mitigation measures and recovery plans that can take into account and counteract emerging inequalities.

## 2 COVID‐19, GENDER, AND HETEROGENEITY IN RURAL LIVELIHOODS

**FIGURE 1:**
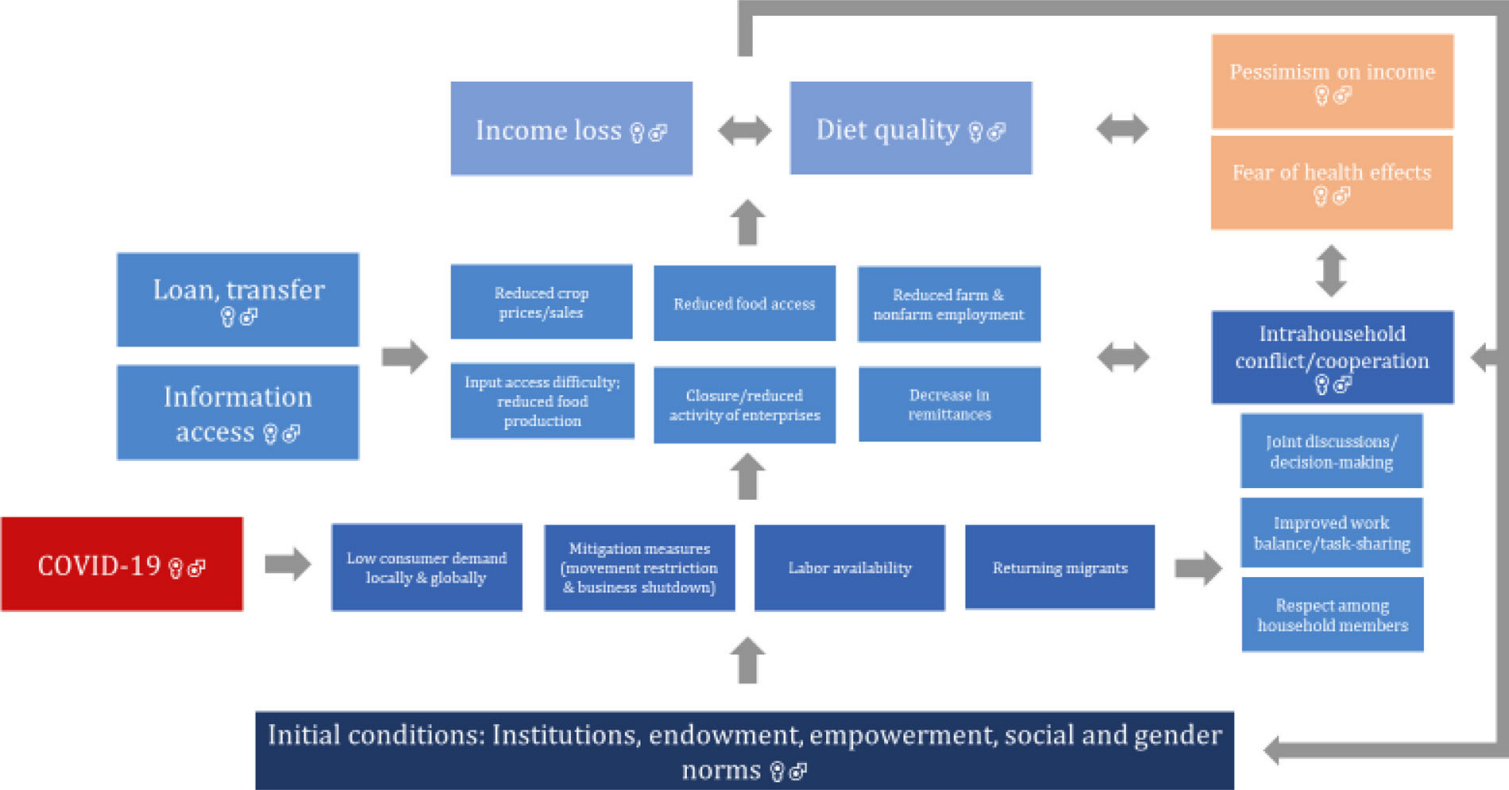
Potential impact pathways of COVID‐19 on rural livelihoods through a gender lens [Color figure can be viewed at wileyonlinelibrary.com]

Even though literature on COVID‐19 is only recently emerging, the anticipated impact pathways of the crisis relate to the wider literature on heterogeneous and gender impacts of crises, shocks, policy interventions, or projects. Figure 1 provides a diagrammatic representation of impact pathways of COVID‐19 on rural livelihoods, with attention to intrahousehold dynamics and gender.

During the first months of the pandemic, a global economic downturn struck as local and national governments established movement restrictions and shut down businesses to contain the spread of the disease. In turn, these factors affected business activities, employment, remittances, and input and output markets. Many households also experienced a significant drop in income and consumption, mitigated through loans or transfers and increased information access (Diao et al., 2020).

The impacts of COVID‐19 will not be uniform across and within households and will depend on households’ assets and wealth; income‐generating activities; access to services, information, loans, and transfers; household composition; and intrahousehold cooperation. In rural areas, we can expect discrepancies between landed households and landless households. However, it should be noted that individuals from landless households also often depend on the agricultural sector for employment as agricultural wage workers or upstream or downstream agricultural entrepreneurs or workers. Farming households, too, often supplement their incomes through nonfarm employment and remittances (Davis et al., 2010).

Even though most countries consider the agricultural sector and food supply chains to be critical, activities in this sector have also been affected by mobility restrictions, shutdowns, and changes in supply and demand (Laborde et al., 2020). Such movement restrictions and resulting employment disruptions will likely affect some more than others during a crisis. As such, gender discrepancies may arise when men and women's employment exhibit distinct characteristics in terms of sectors, roles and responsibilities, and employment conditions (Alon et al., 2020). It is important to maintain women's employment opportunities and income, because they are linked to higher decision‐making power of women and often generate positive outcomes for household welfare (Maertens & Verhofstadt, 2013; Quisumbing et al., 2014, 2015).

Woman‐managed enterprises often have less access to resources, financial services, technical assistance, and market connections, and often experience low survival and productivity rates (Nix et al., 2015; Rijkers & Costa, 2012). Additionally, though use of digital platforms, such as mobile apps, SMS messaging, the internet, and social media, may be more frequent during the crisis, we are likely to see differential access and use, with women and the poorest lagging behind because of lower digital access and literacy (Ragasa & Lambrecht, 2020).

Shocks or crises can reinforce or reduce intrahousehold harmony and intensify domestic violence (Peterman et al., 2020). The COVID‐19 crisis is likely to cause major disruptions in households’ normal daily schedule or setup. These changes may shift household chores, task‐sharing arrangements, childcare or elderly care arrangements, or decision‐making dynamics between women and men within the household. Taking care of the sick falls heavily on women, and school closures have huge implications on the time that women must spend on childcare. Moreover, the sudden impact of COVID‐19 necessitates that many households revise expenditure patterns and forgo major investments. Levels of anxiety and fear may change with the crisis and may vary between women and men.

Last, existing literature notes vulnerabilities, coping mechanisms, and the role of social protection, which may differ between households with different assets and livelihood bases, and between women and men. Studies show that households seeking to cope with a crisis often first sell women's assets such as jewelry (Quisumbing et al., 2013). The use of formal and informal credit facilities may be more frequent during the pandemic and may change gendered control over use of and decisions regarding financial services. In crises, social protection programs are popular policy tools but do not always reach the most vulnerable or consider the specific needs of women (Hidrobo et al., 2020). We explore early evidence on these impact pathways in the context of rural farming communities in Myanmar's Central Dry Zone.

## 3 COVID‐19 IN MYANMAR

Myanmar shares a border of 2,129 km with China, and much of its trade is associated with China. Through border closures, dwindling supply of raw materials for certain industries, and lower demand for services from Myanmar, the COVID‐19 crisis hit the country long before its first infections were confirmed. Remittances from international migration also slowed before lockdowns and other measures aimed at containing the spread of the virus came into effect in Myanmar (Diao & Mahrt, 2020).

At the time of the first round of the phone survey, the reported disease prevalence in Myanmar was relatively low. Myanmar's first case of COVID‐19 was detected on March 23; by May 30, a total of 226 cases had been confirmed and six people had died (MOHS, 2020). On April 6, the Union and regional governments put in place a nationwide stay‐at‐home order (Lwin, 2020). Celebrations for the annual mid‐April Water Festival were prohibited, travel restrictions were put in place, most industrial activities were shut down, public and private administrative services were limited, and all nonessential businesses and schools were closed. These restrictions continued to the end of April and then were gradually lifted. Schools generally resume after the major holidays coinciding with the Water Festival, but COVID‐19 delayed the reopening of schools until at least mid‐July. After a new case of local transmission was found in mid‐August, the number of confirmed cases rose from 375 on August 16 to 3482 cases on September 21 (MOHS, 2020).

Lockdowns and mobility restrictions have affected rural livelihoods. In 2017, nearly one in five rural households in Myanmar received remittance income from either domestic or international migrants (CSO, UNDP, & World Bank, 2020). Because of COVID‐19, many domestic and international migrants can no longer send remittances, thereby reducing income and investment opportunities of rural households (Diao & Mahrt, 2020). Rural businesses also had to shut down for the short or medium term, and residents’ movements were restricted. Phone interviews from May 2020 with agricultural traders in Myanmar's Dry Zone find that three‐quarters of traders faced challenges in both selling and buying crops, lower trading volumes, and lower crop prices (Goeb et al., 2020).

Myanmar has a relatively large landless population in rural areas. In general, landless are poorer than landed households, and a general association exists between landholding and food and nutrition security (Rammohan & Pritchard, 2014). Yet this association depends strongly on linkages with the nonfarm sector. Households with income‐generating strategies in the nonfarm economy typically have better nutritional outcomes than those with income‐generating strategies related exclusively to farming (Pritchard et al., 2019).

Lambrecht and Mahrt (2019) and Winterberger (2017) note a dichotomy in perceptions of gender inequality in Myanmar—among scholars, visitors, and Myanmar's population itself. Myanmar's Gender Development Index (GDI), the ratio of the female to male Human Development Index (HDI) values, was 0.953 in 2018 (UNDP, 2019).^1^ This ratio indicates medium to high equality in HDI achievements between women and men. The country ranks worse than neighboring Thailand but significantly better than Lao People's Democratic Republic, Cambodia, Bangladesh, and India. Because women are active in the public sphere, often managing household income and expenditure, and household members’ perception is that they “co‐own” land and assets and co‐decide on many decisions, many people believe that Myanmar has high gender equality (Akter et al., 2017; Lambrecht & Mahrt, 2019; Winterberger, 2017).

Nevertheless, clear gender discrepancies exist—women are less likely to hold land titles, they play secondary roles in farming and livelihoods, and they face strong gender norms around their role in household chores and domestic care and men's role in farming and entrepreneurship (GEN, 2015; Lambrecht & Mahrt, 2019). Household members generally share their income, and the day‐to‐day household finances are managed by a female household member (Lambrecht & Mahrt, 2019; Winterberger, 2017). Agricultural roles and tasks are defined by gender, with men leading agricultural decision‐making; women rarely participate in information and training activities regarding agricultural production, yet they provide significant amounts of labor on the household farm or as casual farm workers (Carnegie et al., 2020). Ragasa et al. (2020) measure women's and men's empowerment and gender parity using the baseline data of the current phone survey sample. Their results reveal lower empowerment of women than of men; the main contributors of disempowerment are tolerance toward intimate partner violence and women's lack of group membership, work balance, access to and decisions about financial services, respect among household members, and access to agricultural and market information. This article focuses on most of these dimensions and investigates how response measures in the first months of COVID‐19 have shifted these indicators.

## 4 SAMPLE AND METHODOLOGY

### 4.1 Study site

This study focuses on Myanmar's Central Dry Zone, an area with an estimated 12 million people, or about 23% of the total population of Myanmar. The Central Dry Zone covers roughly a third of the country's grain cropping area and includes crops such as pulses, oilseed legumes, sesame, and sunflower. Rice is grown as a rainfed monsoon crop or under irrigation (Herridge et al., 2019). The rainfall pattern in the Dry Zone is bimodal, and the rainfed crop growing season is normally determined by the monsoon season—between May and October—and the dry spell in July (Boutry et al., 2017). Growing rainfed crops has high risks, and access to water for irrigation is critical to reduce risks as well as to expand options available to farmers (Boutry et al., 2017).

**FIGURE 2:**
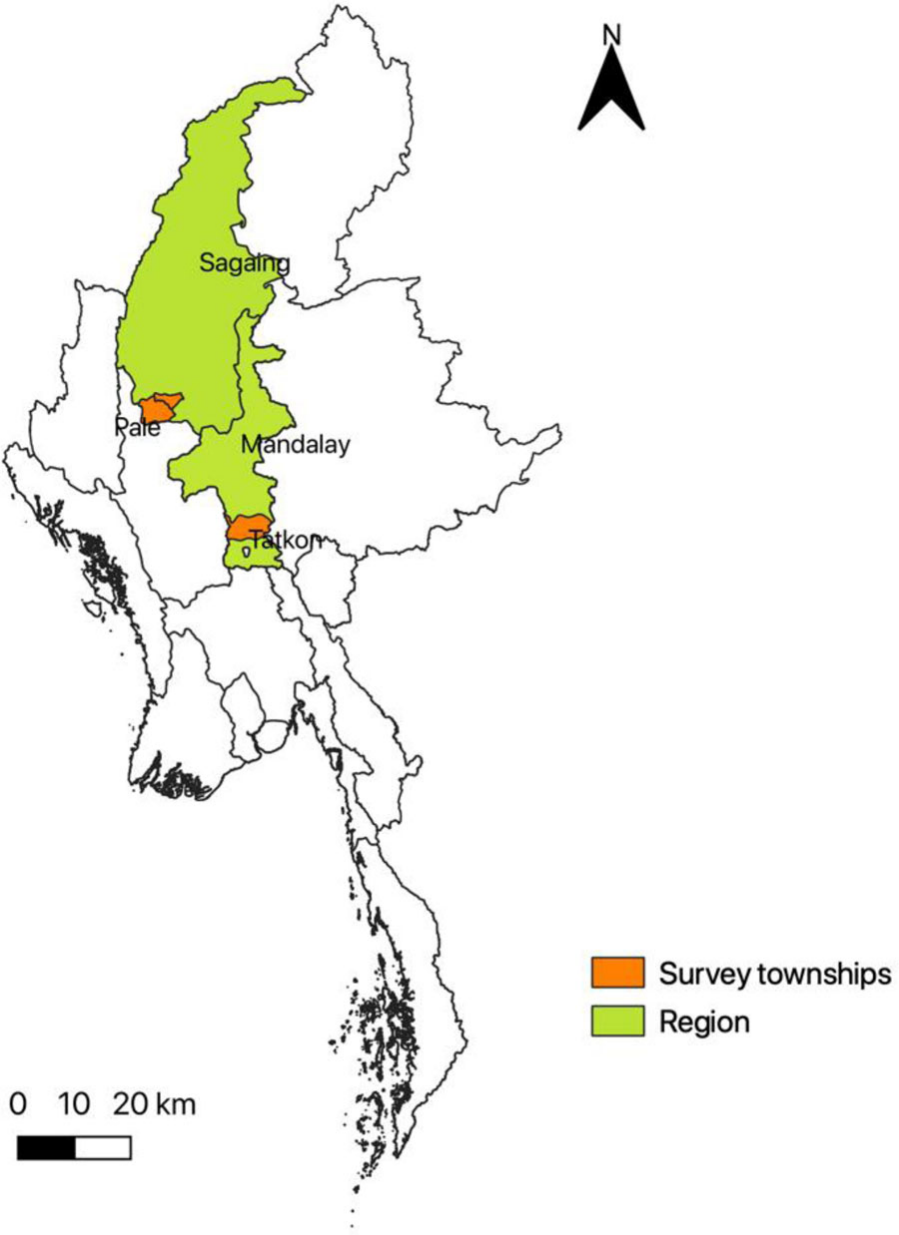
Map of Myanmar and location of the study sites [Color figure can be viewed at wileyonlinelibrary.com] *Source*: IFPRI/World Bank/MSR (2020).

The study communities are located in the catchment areas of two irrigation sites: the Sinthe irrigation site in Tatkon township in Nay Pyi Taw region and the North Yamar irrigation site in Pale and Yinmarbin townships in Sagaing region (Figure 2). They provide a suitable setting to assess the impact of COVID‐19 on irrigated and nonirrigated agricultural production as well as the varied livelihood strategies of landed and landless households in rural communities. The sample communities are relatively homogeneous; most have access to electricity and roads, and are roughly 4–5 km or 20–30 minutes by motorbike from the nearest town center, where household members work in wage or salary employment or sell their produce.

COVID‐19 mitigation measures implemented in the study communities were similar to the measures implemented throughout the country. During April and May, entry into each village was restricted for people from outside the village. Traders and food vendors were not allowed to enter each village but could sell at the village entrance. Family or friends from outside the villages were not allowed to enter, and those recently returning to the villages were required to undergo strict quarantine. Gatherings and social activities were not permitted, and a curfew was implemented at night.

### 4.2 Sampling method and questionnaire

This study builds on an existing baseline survey (BL) conducted in January 2020 before the onset of COVID‐19 (Ragasa et al., 2020). A follow‐up phone survey (PS) was conducted from June 10 to June 17, 2020. Phone surveys with village leaders of four communities were also conducted by one of the authors to inquire about the main COVID‐19 mitigation measures implemented in these communities and about other shocks experienced from February to May 2020.

In total, 1,072 female and male respondents^2^ in 606 landed and landless households in the 30 focus communities were interviewed. Seven percent of the BL households did not have a cellphone, and another 7% did not provide their telephone number during the BL survey. The initial plan was to randomly select a subsample of BL households with a telephone number; however, because 25% of those telephone numbers were not working,^3^ we then contacted all the BL households with telephone numbers. The household attrition from BL to PS was 39%; within the participating households, 6% of individual women and men could not be re‐interviewed. Attrition probit regressions were estimated to identify demographic factors that could explain the likelihood of attrition (Annex Table A1). We used these results to apply inverse probability weighting. The intuition behind this procedure was that inverse probability weighting gave more weight to households that had similar initial characteristics to households that subsequently dropped out than to households with characteristics that made them more likely to remain in the panel. The details are in Annex 1.

The PS averaged 25 minutes per respondent and asked about the immediate impact of the COVID‐19 crisis on households’ incomes, livelihoods, food access and diet quality, remittances and cash and in‐kind transfers, access to social networks and groups, information access, and gender relations and intrahousehold inequality. It covered crop farming activities during the pre‐monsoon season (February–May) at the onset of the crisis and plans for the upcoming monsoon season, which usually starts in June or July. BL indicators tracked during the PS were (1) minimum dietary diversity for women (MDD‐W); (2) household composition; (3) access to information on agriculture, markets, nutrition, or health; (4) receipt of transfers and remittances; and (5) respect among household members (an indicator of intrahousehold harmony).^4^ For indicators for which we had no BL data, we asked respondents to compare conditions and experiences during the crisis (February–May) with those immediately before the crisis (January).^5^ For indicators affected by seasonality, such as farming, time use, employment, nonfarm enterprise, and food consumption, we asked respondents to compare crisis conditions with conditions during the same period last year.^6^

### 4.3 Considerations in the survey design

One potential pitfall in comparing BL and PS values is the change in survey methodology from face‐to‐face to phone interviews. Observed changes of indicators from BL to PS may be partly due to this change in survey methodology. MDD‐W is particularly affected by this difference in method. A rigorous study implemented by Lamanna et al. (2019) in Kenya finds that the MDD‐W data did not change regardless of whether they were collected face‐to‐face or by mobile phone, but researchers used the same survey instrument in that comparison. Following good practice (FAO & FHI 360, 2016), we used a detailed enumeration of all food consumed in the last 24 hours during the face‐to‐face BL. In the PS, however, we used simpler questions requiring only a yes or no answer if the respondent consumed the food groups in the last 24 hours. Nonetheless, we triangulated the data and results with community interviews and other survey questions to determine how much of the observed changes were likely due to COVID‐19 or to other reasons. Because the PS needs to be short and concise, the questionnaire focuses on the experience of the main COVID‐19 impact pathways but falls short in speaking to the depth of these impacts.

Another difference of the PS compared to face‐to‐face interviews is that enumerators cannot observe and ensure the privacy of the interview. If the respondents’ phone is on speaker, for example, it is likely that others in his or her vicinity might overhear the interview. Despite this problem, respondents often prefer to use the speaker phone because it is more comfortable and allows for multitasking during the interview. Enumerators reported at the end of each interview whether or not the phone was on speaker, which they could easily determine from background noise during the call. Additionally, for sensitive questions about intrahousehold relations, respondents were first asked whether they could answer these questions in private.^7^ If not, the module was skipped.

### 4.4 Analytical approach

We focused on descriptive analysis of the household‐level data, disaggregating them by land ownership, irrigation type, and gendered household type. Despite cultural similarities among households, many differences in socioeconomic indicators between irrigation and nonirrigation households and between landed and landless households were observed.^8^ We also analyzed the difference between households with both female and male adults (DHHs) and households with a female adult only (WHHs). At BL, most households comprised a married couple, one or two of their parents or older family members, and either no children or one child. Landed and irrigation households were generally bigger than nonirrigation and landless households. Eighty‐nine percent of the sample households were DHHs, and 11% were WHHs, though household compositions have experienced a slight change due to returning migrants. This change will be discussed in the next section.

Several survey modules asked the primary male and primary female decision‐makers to provide data for comparing women and men and identifying gender gaps. We performed pairwise correlation analysis to check the association of several indicators and estimated regression models to explain variations in indicators at both household and individual levels. In particular, we tested whether asset poverty and deprivation, and indicators at BL were associated with the likelihood of being negatively affected by COVID‐19. We also tested whether empowerment^9^ indicators as well as receipt of remittances, transfers, or information are associated with less negative impacts of COVID‐19. Definitions and descriptive statistics of the household‐ and individual‐level indicators used are displayed in Annex Table A2 and the regressions results are in the other annex tables.

## 5 RESULTS

### 5.1 Farming

Two‐thirds of households in our study area were cultivating crops during the pre‐monsoon season when the COVID‐19 crisis hit. The most commonly grown crop was sesame, but households also grew chickpeas or other crops. Of those farmers cultivating when the pandemic hit, 17% experienced difficulties in purchasing or accessing inputs or services (Table 1). Overall, no difference is observed between DHHs and WHHs. The inputs or services reported as being more difficult to access were agrochemicals (51% of farmers), farm machinery (44%), inorganic fertilizer (39%), and improved seeds (14%).

**TABLE 1:** Difficulties in access to agricultural inputs and markets, February–May 2020 (% of households)

Indicators	All	Irrig.	Nonirrig.	Landed, WHH	Landed, DHH
Difficulty in accessing inputs/services	17	17	15	16	17
Improved seed ^/1^	14	17	0***	10	14
Inorganic fertilizer ^/1^	39	35	62	0	42***
Agrochemicals ^/1^	51	42	100	0	55***
Farm machinery ^/1^	44	52	0	90	41***
Others ^/1^	3	4	0	0	3
Difficulty finding male labor	17	17	14	13	17
Difficulty finding female labor	16	17	14	13	17
Higher wage than normal	22	23	17	22	22
Male laborer this year (MMK/day) ^/2^	5,667	5,686	5,553	6,810	5,572
Male laborer in normal year (MMK/day) ^/2^	4,608	4,617	4,553	4,509	4,615
Female laborer this year (MMK/day) ^/2^	4,806	4,720	4,723	4,564	4,823
Female laborer in normal year (MMK/day) ^/2^	3,975	3,970	4,000	3,342	4,018*
Any difficulties in selling your harvest	66	69	0	76	65
Anticipate any difficulties selling in the following months	34	39	12***	43	33
Will grow crops in the monsoon season	64	96	26***	82	94***
Anticipate difficulty in finding male labor	35	33	46	26	41
Anticipate difficulty in finding female labor	33	41	0***	62	35***
Anticipate difficulty accessing input/services in the coming months	42	42	46	62	46
*Number of households cultivating Feb–May (reporting on difficulty in input access)*	*387*	*371*	*16*	*29*	*358*
*Number of households that intended to sell crops Feb–May (reporting on difficulties in selling)*	*103*	*102*	*1*	*8*	*95*
*Number of households reporting on anticipated difficulties in selling*	*317*	*303*	*14*	*19*	*298*
*Number of households that will cultivate in monsoon (reporting on anticipated difficulties in accessing inputs/services)*					

*Source*: IFPRI/MSR phone survey (June 2020). Irrig. = irrigation households; Nonirrig. = nonirrigation households; WHH = woman‐adult‐only household; DHH = dual‐adult household; N = Number of observations; ^/1^ Percentage among those reporting difficulty in purchasing inputs; ^/2^ Average among those reporting higher wages than usual; Statistically different at.

*** 1%.

** 5%.

* 10% level of significance.

Seventeen and sixteeen percent of cultivating households had difficulty finding and hiring male and female labor, respectively. Twenty‐two percent of households reported higher wages paid for hired labor during the pandemic. For those reporting higher wages paid, increases were 23% for male labor and 21% for female labor. Farm laborers, mostly women, reported less hiring and less work than usual; they therefore experienced greater difficulty in finding farm wage employment.^10^ More than a third of households also anticipated difficulty in finding male (35%) or female (33%) labor, though more WHHs (62%) anticipated difficulties in particular for hiring female labor as compared to DHHs (35%). Forty‐two percent anticipated difficulties in accessing inputs and services during the upcoming monsoon season.

Two‐thirds of farmers experienced difficulty selling their produce, mainly because of lower prices, closed markets, low demand, and movement restrictions. Seventy‐two percent of households had difficulty selling chickpeas, the main crop being harvested and sold when the pandemic hit; among those households, 56% received lower prices. Thirty‐four percent of households anticipated difficulty in marketing their produce in the following months, mostly because of anticipated lower prices. No differences between DHHs and WHHs were observed.

### 5.2 Other livelihoods

Aside from farming, both landed and landless households rely on other sources of income, including wage or salary employment, nonfarm enterprises, and remittances. Both women and men in these households are actively engaged in these activities. Twenty‐two percent of male respondents and 30% of female respondents were usually engaged in farm wage labor (Table 2). Of those usually employed in farm wage labor, 48% of men and 53% of women experienced challenges in finding work during the crisis due to less work than usual, lower pay, or temporary movement restrictions. Thirty‐five percent of male and 30% of female respondents lost their farm wage employment between February and May this year, mostly because of a lack of hiring. For women, additional reasons included household chores and childcare. Sixteen percent of male and 7% of female respondents was usually engaged as nonfarm wage labor. Of those laborers, 68% of men and 49% of women experienced a negative impact on nonfarm work and wages during the crisis, and 31% of men and 57% of women lost their nonfarm wage employment.

**TABLE 2:** Farm and nonfarm wage employment, February–May 2020 (% of respondents)

Indicators	All	W	M	WHH	DHH	Landed	Landless	Landed, W	Landed, M	Landless, W	Landless, M
**Farm wage employment (FWE)**											
Engaged in FWE during normal year	26	30	22***	53	24***	18	44***	20	15*	48	36**
Engaged in FWE this year	21	24	17**	39	19**	13	36**	5	12	41	29*
Experienced difficulty finding FWE ^/1^	51	53	48	46	52	47	55	50	41	55	55
No FWE ^/1^	32	30	35	29	33	32	32	32	33	29	38
Reason for not engaging in FWE											
Nobody to hire me	51	41	65	10	57***	47	54	40	57	41	71
Chores/childcare	10	15	3	40	5*	7	12	7	6	21	0
Poor health/old age	20	26	11	21	20	20	20	22	16	30	6*
Fewer crops to harvest this year	12	13	10	26	9	12	11	13	10	12	11
Not interested/no need to work	9	10	8	18	7	14	5	12	17	8	0
Movement restriction	8	11	4	6	8	11	6	18	0**	5	7
Wages are too low	4	8	0	0	5	10	0	17	0	0	0
Other	5	2	9	0	6	5	5	5	5	0	12
**Nonfarm wage employment (NFWE)**											
ngaged in NFWE during normal year	11	7	16**	8	12	6	22***	3	10***	16	30
ngaged in NFWE this year	8	4	13***	2	8***	5	13**	2	8***	6	23*
Experienced negative impact on work or wages ^/2^	62	49	68	63	62	51	68	69	46	42	85**
No NFWE ^/2^	40	57	31**	82	38**	35	43	47	31	60	31
*Number of observations*	*1072*	*577*	*495*	*140*	*932*	*437*	*495*	*82*	*58*	*140*	*932*

*Source*: IFPRI/MSR phone survey (June 2020). W = women; M = men; WHH = woman‐adult‐only household; DHH = dual‐adult household; ^/1^ Percentage of those normally engaged in FWE; ^/2^ Percentage of those normally engaged in NFWE; Statistically different at.

*** 1%.

** 5%.

* 10% level of significance.

Finally, COVID‐19 affected 67% of female‐led nonfarm businesses and 71% of male‐led nonfarm businesses. No significant difference was observed in the proportion of those affected by COVID‐19 between female‐ and male‐led nonfarm businesses. About half of the businesses (53%) reported having no work at all between February and May.

At BL, 30% of households had relatives working elsewhere and received remittances from those relatives. Thirty‐two percent of landed households and 28% of landless households received remittances. PS data show about 328 returning migrants or new adult additions, affecting 38% of households. Remittances (cash and in‐kind) received during the quarter from February to May averaged MMK 720,000 (USD515), whereas the remittances received during an equivalent 4‐month period at BL averaged MMK 918,669 (USD633), excluding in‐kind remittances not captured in BL. Clearly, households already suffered significant reductions in income due to the loss or reduction in remittances (by at least 20%).

### 5.3 Income loss and coping mechanisms

Fifty‐five percent of households experienced income loss during the crisis (Table 3), with more landless households than landed households affected by income loss (71% versus 48%, respectively). The main coping mechanisms were using savings (78% of households); reducing food expenditure (54%); borrowing, mainly from friends and informal lenders (40%); and selling assets, mainly gold and jewelry (37%). Only one significant difference was observed between DHHs and WHHs: a greater share of WHHs received and accepted a COVID‐19 related transfer from government than DHHs (63 percent vs. 32 percent). On the other hand, additional significant differences were observed between the coping strategies for landless and landed households. More landless households than landed or irrigation households reduced food expenditure.

**TABLE 3:** Income loss, coping mechanisms, and transfers, February–May 2020 (% of households)

Indicator	All	WHH	DHH	Landed	Landless
Decreased income due to COVID‐19	55	50	56	48	71***
Used savings to deal with the reduction in income	78	67	79	77	78
Sold assets to deal with the reduction in income	37	43	36	33	43
Borrowed to deal with the reduction in income	40	50	39	38	43
Reduce food expenditures to deal with the reduction in income	54	48	55	50	62*
Receive and accept a COVID‐19‐related transfer from government	37	63	32***	24	60***
Receive and accept a COVID‐19‐related transfer from an NGO or private individual	7	13	6*	6	7
*Number of observations*	*606*	*69*	*537*	*522*	*84*

*Source*: IFPRI/MSR phone survey (June 2020). WHH = woman‐adult‐only households; DHH = dual‐adult household; N = Number of observations; NGO = nongovernmental organization; Statistically different at.

*** 1%.

** 5%.

* 10% level of significance.

### 5.4 Food access and diet quality

MDD‐W increased in the PS compared to the BL survey (Table 4). Most respondents consumed more diverse foods, with noticeable increases in almost all food groups—especially dark leafy vegetables, Vitamin A–rich fruits and vegetables, nuts, beans, eggs, and dairy. These increases can partly be explained by seasonality. For example, the period covered in our PS coincides with the mango season and postharvest time for pre‐monsoon crops (e.g., chickpeas). Community interviews suggest that increased vegetable consumption results from having more time to harvest wild vegetables as well as from resourcefulness and innovation arising from the crisis coupled with reduced income. An additional explanation could be greater promotion and education regarding nutritious foods from TV, radio, internet, and phone messaging. The community interviews and PS clearly reveal that almost all individuals (women and men) had access to health‐ or nutrition‐related information during the COVID‐19 crisis compared to 47% of respondents having such information before COVID‐19.

**TABLE 4:** Changes in diet quality based on 24‐hour food recall (% of women)

Food group	Baseline	Phone survey	WHH	DHH	Landed	Landless
Grains and roots	100	100	100	100	100	100
Beans	53	75***	79	74	77	71
Nuts/seeds	16	42***	24	45**	45	37**
Dairy	1	3**	3	3	4	0
Meat and fish	77	76	75	77	84	62***
Egg	24	46***	36	47	50	38*
Dark leafy vegetable	61	96***	90	97	96	95
Vitamin A–rich fruits and vegetables	25	86***	90	85	85	87
Other vegetables	89	88	87	88	90	85
Other fruit	26	34	33	34	34	33
**Diet diversity score**	**4.72**	**6.46** ***	**6.18**	**6.50**	**6.65**	**6.10**
*Number of observations*	*997*	*577*	*69*	*508*	*494*	*83*

*Source*: IFPRI/MSR phone survey (June 2020). DHH = dual‐adult household; WHH = woman‐adult‐only household; Statistically different at.

*** 1%.

** 5%.

* 10% level of significance.

We see evidence, however, that the frequency and quantity of meat and fish consumption reduced for many households—by 40% for meat and 28% for fish (Table 5). The main reason for these decreases was lower income. Households experiencing income loss were 24–28% more likely to reduce frequency and quantity of meat and fish consumption during the COVID‐19 crisis (Annex Table A3).

**TABLE 5:** Changes in meat, fish, and vegetable consumption (% of households)

Indicators	Meat and Poultry	Fish	Vegetable
Average number of days in past 7 days of consumption	2.17	3.10	
Compared to a normal year, was this less, more, or the same as you would usually eat this time of year?			
Less	40	28	
More	5	5	
Same	55	67	
Did you eat a smaller quantity than you would usually eat?			
Less	37	25	
More	2	4	
Same	61	71	
Reason for eating less			
Reduced income or money to buy them	79	72	
Not easily available in the market	14	11	
Higher price of fish/meat	19	14	
Afraid to eat too much fish/meat as they may cause more COVID virus transmission	13	10	
Other	10	23	
In last 7 days, did your household eat more orange‐colored vegetables, green leafy vegetables, or other vegetables than you would usually eat this time of year?			
Same as usual			62
Less than usual			2
More orange‐colored vegetables			3
More leafy green vegetables			33
More other vegetables			15

*Source*: IFPRI/MSR phone survey (June 2020).

### 5.5 External support

#### 5.5.1 Transfers

At BL, some households were receiving transfers (cash or in‐kind) from the government. More landed than landless households received transfers at BL (9% versus 4%, respectively). As expected, the proportion of households receiving transfers increased significantly during the crisis: 37% of households received COVID‐19‐related transfers from government, and 7% received transfers from nongovernmental organizations (NGOs) (Table 3). In contrast to BL, more landless households than landed households received transfers during this time (60% versus 24%, respectively). WHHs were more likely to receive transfers related to COVID‐19: 63% of WHHs and 32% of DHHs received government transfers, and 13% of WHHs and 6% of DHHs received transfers from NGOs. Within households, however, most transfers were directed to the household head (often the man), though good practice around the world highly recommends designating women as recipients for cash transfer benefits targeted to a household (see Hidrobo et al., 2020).

Households with poor housing conditions were 14% more likely to get government transfers in the regression models (Annex Table A4). This relationship indicates a good match between the vulnerabilities of the landless or those with dwelling deprivations and measures of poverty. Transfers from NGOs are rare, and it is difficult to see a pattern. Across the different irrigation schemes, we find differences in the likelihood of receiving transfers as well as in the factors related to receiving transfers, such as being landless, having return migrants, or being a WHH.

#### 5.5.2 Information on livelihoods, nutrition, and health

Both women and men received more agricultural and market information during the COVID‐19 crisis than before COVID‐19. During COVID‐19, 55% of women and 65% of men received agriculture or market information, whereas 30% of women and 46% of men received agriculture or market information before COVID‐19. Ninety‐one percent of women and 92% men received information on nutrition or health during COVID‐19, compared to 44% of women and 49% of men receiving such information before COVID‐19. Much of this information promoted awareness about health effects and disease prevention and included promotion of nutritious food. This increase is likely due to the eagerness of both women and men to find information about COVID‐19, its health effects, and the general status of markets and prices, which led to more frequent use of radio, TV, and smartphones. Further survey rounds can investigate whether there is greater cooperation and information sharing within the household.

### 5.6 Stress related to COVID‐19 impacts

Individuals responded differently to questions regarding fear and pessimism about the health and income effects of COVID‐19; responses ranged from being unafraid and very optimistic (0) to being very afraid or very pessimistic (10). In general, women and men were more concerned about impacts on health than about impacts on income (Annex Table A5). No significant difference was observed between women in DHH and WHH or between landless and landed households. Some statistical differences were observed, however, between women and men in DHHs and in landed households. Women in these households were more likely to have greater concerns about health effects of COVID‐19 than were men in those households.

There are some clear associations of pessimism and fear of COVID‐19 effects and gender, age, education, household type, location, empowerment, and receipt of transfers and information (Annex Table A6). Men were more pessimistic about income effects and less fearful about health effects than were women. This finding coincides with gender stereotypes of men earning income and women performing more care responsibilities. Older people were less fearful about health effects. People with no formal education were more optimistic about the income effect than were those with more years of formal education.

Respondents in WHHs were less likely to be fearful about health effects, less pessimistic about income effects, and less likely to be too optimistic or too pessimistic. Those in landed households were less likely to be very fearful and the nonpoor (no dwelling deprivation) were less likely to be fearful of health effects and less likely to be pessimistic about income effects.

Those in households receiving government transfers were less likely than those not receiving transfers to be fearful of the health effects of COVID‐19. Households receiving transfers from NGOs were more likely to be optimistic than were those not receiving transfers. More‐empowered respondents were more likely to be optimistic about COVID‐19′s income effect in some models. Those in larger households were more likely to be pessimistic than were those in smaller households.

### 5.7 Intrahousehold dynamics

#### 5.7.1 Household composition

In 41% of the households, respondents reported changes in household composition compared to the household roster at BL. These changes mostly included additions of household members. Eighty‐two percent of new household members returned from migration, 6% returned home from school, and others joined for various reasons. Interestingly, with the return of many migrants, a third of households (24 observations) originally classified as WHHs during BL reported adult men as household members during PS. Note that in the main analyses in this article we follow the BL classification of these households.

Annex Table A7 shows the changes in indicators in these new DHHs, as compared to the other households. In these new DHHs, women achieved less work balance and worked more hours than did women in DHHs and WHHs during BL. Additionally, significantly more women in these new DHHs reported increased time spent on all activities (chores, care, farm and nonfarm work) in the PS than WHHs during BL and significantly less women in these new DHHs reported decreased time spent in different activities than did women in DHHs (Table A7). A total of 50% of women in these new DHHs reported an overall increase in time spent across all work during COVID‐19. Women in these new DHHs were less likely to report a decrease in time spent in various activities during COVID‐19 compared to usual than were women in other household types. Interestingly, the share of women in new DHHs who achieved adequacy in respect among members worsened in the PS, whereas female respondents in WHHs improved in this indicator. Moreover, more women in new DHHs reported more stress or tension in the household during COVID‐19 than before COVID‐19 as compared with women in other household types.

#### 5.7.2 Work balance and task‐sharing

Annex Table A8 shows the hours spent per activity grouping during January (BL survey).^11^ In landed households at BL, a total of 27% of women and 19% of men reported a heavy workload and did not achieve work balance (> 10.5 hours of work). Within landless households, a total of 36% of women and 22% of men reported a heavy workload. These workload estimates are likely worse in the planting weeks during the monsoon season (the PS survey). Women in landless households are particularly more vulnerable in terms of workload. Despite a clear dichotomy of tasks within landed households (women and men work together, but women focus on chores and care and men focus on farm work), women in landless households usually spend the same time as men in farm and nonfarm work, and also carry most responsibility for chores and care.

There are some indications that time burden has worsened with COVID‐19: 38% of women and men respondents reported overall increased time spent across various activities during COVID‐19 compared to usual (Table 6). A total of 39% of women and 35% of men reported increased time spent on chores and care.^12^ Slightly more women reported increased time spent on household chores and care. Most respondents did not report a change in time spent on farming; for those who reported a change, more men reported an increase and more women reported a decrease. In landed households, most respondents reported no change in time spent on nonfarm work. In landless households, however, we see more decreases. Almost two‐thirds of women in landless households reported no change, almost a fourth reported a decrease, and the remaining 15% reported a increase; in contrast, 42% of men in these households reported decreased time spent on wage and nonfarm enterprises. Overall, more men than women reported decreases in time spent on wage or nonfarm enterprises—because of less work or business closure (Table 6). For men, there seems to be some shift of time and tasks from wage and nonfarm work to household chores and care. It is less clear why many women reported an overall increase in time spent across household, farm, and nonfarm work. Although individuals may have more available time to perform chores and care because of less time spent on livelihood activities, this seems not to be the case for many women, with several even reporting not engaging in wage employment because of household chores and care during COVID‐19. Women, especially in landless households, are most vulnerable because they had a heavier workload to start with at BL. A total of 37% of women in landless households reported an overall increase in time spent across various activities during COVID‐19 compared to usual.

**TABLE 6:** Change in time spent in household and employment activities by individuals (% of respondents)

	All households			Landed households			Landless households			Gap	
	Women	Men	Gender gap	Women	Men	Gender gap	Women	Men	Gender gap	Women (Landed vs. Landless)	Men (Landed vs. Landless)
**Household chores and care**											
Less than before	7	4	–3**	6	4	–2	9	3	–6*	–2	1
About the same as before	54	61	7*	54	61	6	53	61	8	1	–1
More than before	39	35	–3	39	35	–4	38	36	–2	1	–1
*N*	*577*	*495*		*494*	*435*		*83*	*60*			
**Farm work**											
Less than before	7	6	–2								
About the same as before	87	77	–10***								
More than before	5	18	12***								
*N*	*495*	*437*									
**Wage and nonfarm enterprise work**											
Less than before	19	28	9	17	17	1	22	42	20	–6	–25**
About the same as before	71	66	–4	77	78	1	63	51	–12	14*	28***
More than before	10	6	–5	6	5	–2	15	7	–8	–8	–3
*N*	*254*	*224*		*208*	*187*		*46*	*37*			
**Overall change in household, farm, wage, and enterprise work**											
Less than before	11	10	–1	11	9	–3	12	15	2	–1	–6
About the same as before	51	51	0	51	51	0	51	51	0	0	0
More than before	38	38	1	38	40	2	37	34	–3	0	6
*N*	*577*	*495*		*494*	*435*		*83*	*60*			

*Source*: IFPRI/MSR phone survey (June 2020). Statistically different at.

*** 1%.

** 5%.

* 10% level of significance.

We define improved household task‐sharing as a scenario in which the male respondent's time spent on care and chores increased and the female respondent's time spent on these activities remained the same or decreased. Eighteen percent of households showed evidence of improved task‐sharing in childcare, 13% showed evidence of improved task‐sharing in elder care, and 12% showed evidence of improved task‐sharing in household chores (Annex Table A9). We did not see clear patterns of households with improved task‐sharing, except that larger households (more members) were more likely to experience task‐sharing.

A probit model on the relationship of reported increases in time spent on care and chores during COVID‐19 and work balance during BL shows that those achieving work balance and with a lower workload in BL were less likely to report increases in time spent on household chores and care during COVID‐19; in contrast, those not achieving work balance and with a higher workload in BL were more likely to report increases in time spent (Annex Table A10). Interestingly, those with a higher empowerment score were more likely to report decreases in time use during COVID‐19 compared to usual. These relationships are only correlates, and further in‐depth research will be needed to understand these dynamics of time use and task‐sharing.

#### 5.7.3 Decision‐making and intrahousehold harmony

More discussions on expenditures and coping strategies within the household were reported. Nevertheless, most women and men reported having the same level of input in decision‐making as before COVID‐19, but more women reported having greater input in decision‐making than usual compared to men (Annex Table A9).

Most respondents rarely had a disagreement with their spouse or partner in the 2 weeks before the survey (early June), whereas 10 percent of women and men in landed households and 22 percent of women and 11 percent of men in landless households said they often or sometimes had a disagreement (Table 7). Most women and men had similar tension at home during the COVID‐19 crisis as before, whereas 14 percent women and 17 percent men in landed households and 24 percent of women and 21 percent of men in landless households experienced more tension. Women in landless households more often reported disagreement in the previous 2 weeks and more than usual tension at home during COVID‐19 compared to men in landless households and other household types.

**TABLE 7:** Intrahousehold tension or conflict (% of respondents)

	Landed households			Landless households			Gap	
	Women	Men	Gender gap	Women	Men	Gender gap	Women (landed vs. landless)	Men (landed vs. landless)
In your relationship with your partner, how often would you say that you had a disagreement or fought in the last 2 weeks?								
Rarely	90	90	0	78	89	11*	12**	–1
Sometimes/Often	10	10	0	22	11	–11*	–12**	–1
								
*N*	472	428		69	58			
Is the COVID‐19 crisis situation causing more or less tension/stress/conflict in the household than usual?								
The same	82	76	–6*	72	74	2	10	2
More stress than before	14	17	3	24	21	–3	–10	–4
Less stress than before	5	7	2	4	6	1	0	1
*N*	474	429		70	60			

*Source*: IFPRI/MSR phone survey (June 2020). Statistically different at.

*** 1%.

** 5%.

* 10% level of significance.

We looked at another measure of intrahousehold harmony. In BL and PS, more men than women achieved adequacy in respect among household members (Table 8). During PS, 75% of women and 79% of men in landed households and 56% of women and 72% of men in landless households achieved adequacy in respect. Women in landless households were the least likely to achieve adequacy in respect. Although the majority of women and men reported no change from BL to PS, more women than men reported decreased adequacy in respect among household members from BL to PS (20% of women versus 15% of men), and this difference is statistically significant. Therefore, we see a worsening gender gap in this indicator from BL to PS. In terms of household‐level changes from BL to PS, 51% of households had no change in this indicator for both female and male respondents, 20% saw some improvement for at least one respondent (without any respondent experiencing a worsened indicator), and 29% saw at least one respondent worsening in this indicator. We see heterogeneity across households in terms of changes in respect and intrahousehold harmony. Overall, though, one should be cautious because part of this change might be due to the change from face‐to‐face to PS interviews.

**TABLE 8:** Respect among household members

(a) By survey round (% of respondents)									
	Baseline			Phone Survey			Gap		
	Women	Men	Gender gap	Women	Men	Gender gap	Women (BL vs. PS)	Men (BL vs. PS)	
Respondents achieving adequacy in respect among HH members	72	78	6**	69	77	8**	–3	–1	
*939*	939	855		544	489				

(b) By land ownership, during PS (% of respondents)									
	Landed households			Landless households			Gap		
	Women	Men	Gender gap	Women	Men	Gender gap	Women (Landed vs. Landless)	Men (Landed vs. Landless)	
Respondents achieving adequacy in respect among HH members	75	79	5	56	72	15*	18**	8	
*N*	474	429		70	60				

(c) By individual change from BL to PS (% of respondents)									
	Women (N = 544)			Men (N = 489)			Gender gap		
	Improved	Worsened	No change	Improved	Worsened	No change	Improved	Worsened	No change
Respondents achieving adequacy in respect among HH members	16	20	64	12	15	75	–4	–5**	9**

(d) By intrahousehold inequality in respect among household members (% of households)									
	Baseline (N = 855)			Phone survey (N = 460)			Change (N = 460)		
	W > M	M > W	W = M	W > M	M > W	W = M	Improvement (no decline for both)	Worse for any W/M	Same for both W&M
Respondents achieving adequacy in respect among HH members	10	16	74	11	18	71	20	29	51

*Source*: IFPRI/MSR phone survey (June 2020). M = men; W = women; Statistically different at.

*** 1%.

** 5%.

* 10% level of significance.

Some clear associations exist between reported intrahousehold harmony and gender, age, location, household type, and receipt of transfer (Annex Table A6). Men were more likely to achieve adequacy in respect among household members. Men were more likely than women to report less stress than usual within the household during COVID‐19. Older people were less likely than younger people to report more discussion about income use and coping mechanisms in the household during COVID‐19 than before, and to report less input to discussions and decisions during COVID‐19. Older people were less likely than younger people to report more stress within the household.

Respondents in the second irrigation site were more likely to report more stress than usual than were those in the other site. Respondents in WHHs were more likely to report more stress during COVID‐19 than before. Those in landed households were more likely to have achieved respect among household members and were less likely to report less discussion within the household during COVID‐19 than before. Those with income loss were more likely to report more stress and more frequent disagreements within the household during COVID‐19 than before. Those in households receiving transfers from the government were more likely to achieve adequacy in respect. Those receiving more agriculture or market information were more likely to report more discussions in the household during COVID‐19 than before.

## 6 CONCLUSIONS

The COVID‐19 crisis has affected people around the world through several pathways and to different extents. In rural areas, we can expect households and members within these households to be affected differently according to initial conditions such as assets, wealth, and livelihood strategies. This article analyzed the heterogeneous and gender‐differentiated impacts of COVID‐19 on rural livelihoods and poverty, nutrition, and intrahousehold relations in Myanmar's Central Dry Zone, building on baseline data collected in January 2020 and phone survey data collected in June 2020 from 1072 female and male respondents.

Our results confirm that rural communities have been heavily and heterogeneously affected by the COVID‐19 crisis. All rural livelihood sources were affected during the onset of the COVID‐19 crisis: farming, nonfarm enterprises, wage employment, and remittances. Roughly 17% of farming households experienced difficulties in accessing inputs, and 66% that had harvested and were selling when the pandemic hit were affected by lower prices and lower demand. About half of farm workers usually employed as farm wage labor and almost two‐thirds of those usually employed as nonfarm labor experienced difficulty finding work during the crisis. About 68% of all businesses were affected by COVID‐19 through reduced activity and lower demand. More than a third of all households experienced the return of migrant household members, and households on average received at least 20% less in remittances.

These factors resulted in reduced income from all livelihood sources. Four months after the onset of the COVID‐19 crisis, over half of the survey households experienced income loss due to COVID‐19, though some households were more severely affected than others. Landless households were more often affected by income loss and other disruptions caused by COVID‐19 than were landed households. Women and men in these landless households were equally engaged and affected by lower wages or more difficulties in finding farm work; fewer women were engaged in nonfarm work, but almost all of them lost this nonfarm wage employment. We do not see distinct and major differences in the proportion of DHHs and WHHs or men and women affected by COVID‐19. Nonetheless, more WHHs than DHHs experienced greater difficulties in accessing farm machinery services and anticipated difficulties in finding and hiring labor for the monsoon season.

Despite no reported shortages of food, over half of all households experiencing decreased income reduced food expenditures as a coping mechanism. Surprisingly, diets were more diversified, largely due to seasonality and, to some extent, survey method change. This diversity is also likely attributable to the intensified promotion and education by the government and NGOs about nutritious foods to fight COVID‐19. Access to information related to agriculture, market, nutrition, and health seems to have improved during COVID‐19 for both women and men, and the gender gap in information access within the household has slightly reduced. Although rigorous assessment is needed to substantiate this effect, this finding points to positive and hopeful signs of what governments can meaningfully achieve through information campaigns.

Aside from reducing food expenditures to deal with income losses, 40% of households with dereased income had borrowed from friends or informal lenders, and 37% had already sold assets such as gold, land, or jewelry. Over a third of households also received government transfers to cope with the COVID‐19 crisis, and 7% received transfers from NGOs or individuals. These transfers were targeted to more vulnerable households: landless households and households with poor housing conditions were more likely to be recipients of government transfers, providing some hopeful signs of targeting toward these households. Interventions and transfers did not specifically target women, however, and households in some locations were less likely to get transfers, indicating a possible opportunity to improve geographical balance and inclusion in social protection programs.

Our study does not observe major changes in gender divisions and gaps, although we highlight here some interesting patterns. In line with women's and men's perceived roles at home, women are more likely to worry about the health impacts of COVID‐19, although women and men equally are concerned about the income impacts. Despite the negative impacts on earnings and employment, there is no evidence that time and task allocation changed significantly, and gender gaps have remained—or even worsened for some. Women were more likely than men to report increases in workload related to household chores and care during COVID‐19. Women in landless households are particularly vulnerable in terms of increased workload, worsened respect, and increased tension in the household during COVID‐19. Despite a clear dichotomy of tasks within landed households (women and men work together, but women focus on chores and care while men focus on farm work), women in landless households spend as much time as men do on farm and nonfarm work but also have to carry the main responsibility for chores and care. This time burden seems to have worsened during COVID‐19, and future research can explore this area more extensively. Empowerment scores at BL did not show significant correlations on COVID‐19 impacts, except on the optimism level and change in time use—that is, those where were empowered at BL were more likely to report decreased time spent on household work. This is another area for further investigation.

Given that our sample communities are neither among the poorest nor among the worst affected in the country, and given that this crisis has only begun, our findings are deeply worrying. Continued efforts are needed to ensure that the agricultural sector, critical to food and nutrition security, can function safely and optimally, including the trade of agricultural products. The main disruption for the farming households was on the marketing of their produce. In the short‐term, collective and innovative marketing arrangements can be promoted, along with assistance in storage and processing practices. These efforts include continuing to promote trader e‐commerce, which could reduce in‐person interactions, and avoiding closures or reducing operating hours of markets or trading centers, which risks overcrowding of facilities. In the medium and long term, the government and partners can be push for a more aggressive strategy to mobilize demand and expand market opportunities for agricultual produce. The increased loan amount per acre provided by MADB^13^ as well as provision of loans to small and medium enterprises will help soften the impact on agricultural production, though whether this mitigation is sufficient to ensure that agricultural production and incomes are on par must continue to be monitored so that swift action can be taken when needed. Myanmar's farmers are vulnerable not only to shocks related to COVID‐19 but also to climatic shocks such as droughts and floods.

Where possible, short‐term loan programs and input packages, as well as government transfers, should explicitly target women producers and entrepreneurs. The past Maternal and Child Cash Transfer program in Myanmar has demonstrated that transferring money to women is a feasible and successful approach to increase the share of the household budget under women's control (Maffioli et al., 2019). Putting money in women's hands is also highly recommended as a good practice in many countries (Hidrobo et al., 2020).

Information campaigns regarding health and nutrition impacts are effectively reaching rural households. Given that we continue to observe similar (or even worsened) gender gaps in workload and task sharing, and increased reporting of discussions and arguments at home, such information campaigns could be expanded to include gender awareness and to promote intrahousehold harmony and more equal task‐sharing at home.

Our findings show rich and rigorous information on the impacts of COVID‐19 in rural communities in Myanmar's Dry Zone, yet our work entails significant shortcomings such as a limited geographical spread. The recommended short duration of phone surveys and other disadvantages compared to face‐to‐face interviews also limit our insights into the depth of the impact of the COVID‐19 crisis. Moreover, the crisis has not yet come to an end.

More research and follow‐up surveys are needed for continued monitoring of the medium‐ and long‐term effects of COVID‐19 and of its heterogeneous impact across households and among men and women. Subsequent analysis and data collection can help explore more details on changes in time use, task and information sharing in households, and how effective intrahousehold cooperation might help mitigate negative impacts of this crisis. In particular, changes in household composition and whether they positively or negatively affect the welfare of different household members will be an important research question. Questions also remain on the differential impact of targeting of social transfers particularly to women—rather than to men who are generally considered the head of household—and to what extent the modality and size of transfers might help attenuate adverse impacts of COVID‐19 such as loss of assets or reduced food expenditures. Last, as a second wave of cases is observed and mobility restrictions are put in place in the country after this survey round, it is important to continue to monitor the impacts of these new measures.

## Supporting information

Table A1. Attrition probit estimates (unrestricted regression, equation 1)
Table A2. Definition and descriptive statistics (mean) of household‐ and individual‐level indicators used
Table A3. Correlates of income loss, food consumption, and intrahousehold gaps in respect
Table A4. Correlates of receipt of transfer
Table A5. Level of concern about COVID‐19 (% of respondents)
Table A6. Correlates of individual level of stress, pessimism and fear of COVID effects
Table A7. Comparison of new DHH to other groups
Table A8. Reported hours spent on various activities (24‐hour recall)
Table A9. Change in time spent in household and employment activities and change in inputs to decision‐making within household (% of households)
Table A10. Correlates of time use change during COVID

Supplement Material

## Footnotes

1 HDI is a summary measure of average achievement in key dimensions of human development: life expectancy at birth, mean years of schooling for adults and expected years of schooling for children, and gross national income per capita. It does not measure other relevant aspects of empowerment commonly measured in the gender literature, such as decision‐making power. The GDI is the ratio of the HDIs calculated separately for women and men using the same methodology as in the HDI.

2 We interviewed one primary female decision‐maker and one primary male decision‐maker (for decisions on livelihoods), consistent with the selection of two respondents (of opposite sex) as in the Women's Empowerment in Agriculture Index (WEAI) methodology (Malapit et al. 2019). In our sample, these decision‐makers are mainly couples (husband and wife). In the few households with no couples, the female adult daughter and her father or a male adult member of the household were interviewed. We interviewed the same individuals in both the BL and the PS.

3 The Government of Myanmar ordered mandatory registration of SIM cards by June 30, 2020. This order likely led to a large share of phone numbers being blocked and users changing phone numbers. (https://www.irrawaddy.com/news/burma/myanmar‐mobile‐users‐told‐re‐register‐sim‐cards‐june‐30.html)

4 “Respect among household members” is one of the 12 indicators of the project‐level WEAI (or pro‐WEAI) methodology (Malapit et al. 2019) and a measure of intrahousehold harmony (see Annex Table A2 for the measurement). It is among the top contributors of disempowerment among women and men and a source of gender gap identified during BL. This and a more direct question about change in tension, stress, or conflict within the household are used as early indicators for domestic violence, about which it is very difficult to collect data but which is a commonly hypothesized negative outcome of the COVID‐19 crisis (see Peterman et al. 2020).

5 For example, we asked, “In February, March, April, and May, did you use the internet or use social media on your cell phone more than usual (before COVID‐19 crisis)?”

6 For example, we asked, “During February, March, April, or May, did you spend more hours than you would normally do this time of year on caring for the children?”

7 The enumerator asked, “Now I would like to ask you some questions about how you feel about one other person in your household or family group and how you think he or she feels about you. Your responses will be confidential if nobody is listening with you to this interview. Is it possible for you to answer these questions in private?” Six respondents said they could not respond in private: five men and one woman, and for them the module on intrahousehold dynamics was skipped.

8 Nonirrigation households largely overlap with landless households; only 5% of landed households did not have irrigation access. In our findings most results are similar when disaggregating between landless and landed as compared to between irrigation and nonirrigation households.

9 We adopted the pro‐WEAI, a survey‐based tool that aggregates women's and men's achievements across 12 equally weighted indicators that measure three types of agency: intrinsic, instrumental, and collective (see Malapit et al. 2019).

10 These responses suggest a contradiction: difficulty in hiring labor or the need to pay higher wages from the landed households’ perspective on the one hand and difficulty in finding employment from the hired laborer's perspective on the other. One part of the story is the heterogeneity of the farmers’ experiences in the focus communities. A second part of the story is that some farmers had less money to hire labor (because of reduced incomes); thus, they were less likely to hire labor and therefore reported difficulty in hiring labor or in paying higher wages. This second story is consistent with that of hired labor having difficulty finding farm wage employment.

11 Roughly half of cropland is cultivated during the post‐monsoon season, and January is harvest time for the post‐monsoon season or planting time for the pre‐monsoon season. In general, farmers are less busy during January (the BL survey) than during May (the PS survey), so workload reported during PS will likely be greater.

12 Respondents were asked the following questions: “During February, March, April, or May, did you spend more hours than you would normally do this time of year on caring for the children?”; “During February, March, April, or May, did you spend more hours than you would normally do this time of year on caring for the elderly?”; and “During February, March, April, or May, did you spend more hours than you would normally do this time of year on household chores such as cooking, cleaning, washing, etc.?”

13
https://elevenmyanmar.com/news/ks‐600‐billion‐earmarked‐for‐covid‐19‐special‐loan‐for‐farmers; https://www.mmtimes.com/news/almost‐three‐quarters‐covid‐19‐fund‐myanmar‐distributed‐smes.html.
