# Supplementary material for: Immediate impacts of COVID‐19 on female and male farmers in central Myanmar: Phone‐based household survey evidence
Source: Agric Econ. 2021 May 3;52(3):505–23. doi: 10.1111/agec.12632 (PMC8207076; doi:10.1111/agec.12632)
Supplement: Supplementary file 1 — Table A1. Attrition probit estimates (unrestricted regression, equation 1) Table A2. Definition and descriptive statistics (mean) of household‐ and individual‐level indicators used Table A3. Correlates of income loss, food consumption, and intrahousehold gaps in respect Table A4. Correlates of receipt of transfer Table A5. Level of concern about COVID‐19 (% of respondents) Table A6. Correlates of individual level of stress, pessimism and fear of COVID effects Table A7. Comparison of new DHH to other groups Table A8. Reported hours spent on various activities (24‐hour recall) Table A9. Change in time spent in household and employment activities and change in inputs to decision‐making within household (% of households) Table A10. Correlates of time use change during COVID [file AGEC-52-505-s002.docx]

**Annex 1. Testing and addressing attrition from baseline data collection to phone survey**

There are three sources of household attrition from baseline survey (BL) to phone survey (PS): the household did not have smartphone, it did not give a telephone number during BL, or the telephone number was not working. Seven percent of the sample households did not have a smartphone. Another 7 percent did not provide telephone numbers (even though they reported owning at least one cell phone during BL). Of those with telephone numbers, 75 percent were successful interviews and 25 percent had nonworking telephone numbers or no answer despite seven attempts, and less than 1 percent did not give consent for the interview. The total household attrition from these various causes is 39 percent. Table A1 shows the determinants of attrition by the cause of attrition. Households in Yinmabin township were more likely to drop out than were those in other townships (Table A1, column 4). Dual-adult households were more likely than women-adult-only households to drop out. Those in the two richest quintiles were less likely to drop out than were those in the lower (poorer) quintiles according to a principal component analysis of various assets owned by the household.

Within the participating households, there is also attrition at the individual level (because we interviewed both female and male decision-makers within the household). Six percent of the female and male respondents dropped out in the PS (Table A1). Those in Tatkone were more likely to drop out than were those in other townships (Table A1, column 5). The relationship of age and attrition is nonlinear—the youngest and oldest were less likely to drop out, but those in the middle were more likely to drop out. Those with higher education and those receiving transfers were less likely to drop out. Those in richer households (measured in terms of dwelling deprivation) were more likely to drop out.

The first step conducted was to test whether attrition in a panel data model is random. We used attrition probit regressions (Fitzgerald et al. 1998) and checked the significance of the explanatory variables and pseudo R-squared, which can be interpreted as the proportion of attrition that is nonrandom (Outes-Leon and Dercon 2008). The pseudo R-squared is 8 percent in the household-level attrition probit and individual-level attrition probit, which can be interpreted as the proportion of attrition that is nonrandom.

We addressed attrition by applying an inverse probability weighting. The intuition behind this procedure is that it gives more weight to households that have similar initial characteristics to households that subsequently drop out than to households with characteristics that make them more likely to remain in the panel (Baulch and Quisumbing, no date; World Bank 2020). First, we constructed a variable in the baseline dataset which denotes if the household responded in phone survey. This variable is the dependent variable in the regression. Second, we performed a logistic regression model to determine the likelihood of non-response or attribution based on the household and/or individual characteristics (Table A1). Third, we divided the continuous measure of the likelihood of attrition into deciles and collapse to the mean and applied them as non-response adjustment. These steps are also similar to estimating the logistic regression of successful interviews in phone surveys and taking the reciprocal of their collapsed means by decile groups and applied them as the non-response adjustment. Lastly, we double-checked to make sure that this non-response adjustment is greater than 1 for those respondents that had higher levels of non-response (i.e. the value of the weights is increasing to compensate for those with similar characteristics but did not respond).

References

Baulch, B., & Quisumbing, A. (No date). Testing and adjusting for attrition in household panel data. Chronic Poverty Research Centre (CPRC) Toolkit Note.

Fitzgerald, J., Gottschalk, P., & Moffit, R. (1998). An analysis of sample attrition in panel data. *Journal of Human Resources,* 33(2), 251–99.

Josephson, A, Kilic, T, & Michler, J. 2020. Socioeconomic impacts of COVID-19 in four African countries. World Bank Research Policy Paper 9466.

Outes-Leon, I., & Dercon, S. (2008). Survey Attrition and Bias in Young Lives. Young Lives Technical Note 5. University of Oxford, Oxford.

World Bank. 2020. High Frequency Mobile Phone Surveys of Households to Assess the Impacts of COVID-19 Guidelines on Sampling Design. Accessed on March 13, 2021 on <http://documents1.worldbank.org/curated/en/742581588695955271/pdf/Guidelines-on-Sampling-Design.pdf>

**Table A1. Attrition logistical regression estimates**

| Explanatory variables | (1) | (2) | (3) | (4) | (5) |
| --- | --- | --- | --- | --- | --- |
|  | No smartphone (=1) | No telephone number (=1) | Nonworking telephone number (=1) | Total household attrition from BL to PS (=1) | Individual-level attrition (=1) ^/a^ |
| Township (reference=Pale) |  |  |  |  |  |
| Tatkone | 0.435** | 0.262* | 0.035 | 0.117 | 0.402*** |
|  | (0.203) | (0.139) | (0.155) | (0.114) | (0.119) |
| Yinmabin | 0.407 | 0.370*** | 0.547** | 0.535** | -0.021 |
|  | (0.271) | (0.092) | (0.262) | (0.222) | (0.202) |
| Dual-adult HH (=1) | -0.667** | -0.097 | 0.607** | 0.431** |  |
|  | (0.315) | (0.237) | (0.246) | (0.207) |  |
| Irrigation HH (=1) | -0.138 | -0.027 | 0.220 | 0.113 | 0.232 |
|  | (0.219) | (0.241) | (0.293) | (0.232) | (0.203) |
| Participates in farming activity (=1) | 0.673** | -0.326 | -0.353 | -0.300 | -0.221 |
|  | (0.322) | (0.368) | (0.292) | (0.245) | (0.263) |
| Male (=1) ^/b^ | 0.609* | 0.104 | -0.360* | -0.239 | -0.110 |
|  | (0.333) | (0.221) | (0.208) | (0.181) | (0.157) |
| Age | -0.009 | -0.024 | 0.010 | -0.005 | -0.083** |
|  | (0.043) | (0.040) | (0.029) | (0.026) | (0.033) |
| Age squared | 0.000 | 0.000 | -0.000 | 0.000 | 0.001** |
|  | (0.000) | (0.000) | (0.000) | (0.000) | (0.000) |
| *Education level (reference=no formal schooling)* |  |  |  |  |  |
| Primary school | -0.117 | -0.115 | 0.167 | 0.124 | -0.271** |
|  | (0.171) | (0.149) | (0.116) | (0.115) | (0.132) |
| High school | -0.285 | -0.463** | 0.143 | 0.029 | -0.749*** |
|  | (0.357) | (0.230) | (0.210) | (0.201) | (0.284) |
| *Literacy level (reference=cannot read or write)* |  |  |  |  |  |
| Can read and write | 0.146 | -0.298 | 0.295 | 0.081 | 0.400 |
|  | (0.360) | (0.260) | (0.331) | (0.264) | (0.449) |
| Fluent | 0.076 | -0.449* | 0.136 | -0.154 | 0.463 |
|  | (0.317) | (0.251) | (0.339) | (0.259) | (0.513) |
| *Marital status (reference=single)* |  |  |  |  |  |
| Married | -0.733*** | -0.174 | 0.315 | 0.152 | -0.288 |
|  | (0.176) | (0.248) | (0.320) | (0.245) | (0.218) |
| Widow/Widower/Divorced | -0.696** | -0.082 | 0.331 | 0.200 | -0.069 |
|  | (0.273) | (0.234) | (0.308) | (0.238) | (0.340) |
| *Main occupation (reference=no job)* |  |  |  |  |  |
| Farmer (own land) | -0.018 | 0.338 | 0.191 | 0.250 | 0.188 |
|  | (0.258) | (0.300) | (0.171) | (0.167) | (0.303) |
| Agricultural labor | 0.655* | 0.362 | 0.063 | 0.227 | 0.265 |
|  | (0.352) | (0.368) | (0.291) | (0.245) | (0.337) |
| Other jobs | 0.113 | -0.066 | -0.319 | -0.289 | 0.128 |
|  | (0.310) | (0.333) | (0.223) | (0.224) | (0.350) |
| Household size | -0.318*** | -0.014 | -0.025 | -0.022 | -0.021 |
|  | (0.068) | (0.037) | (0.028) | (0.022) | (0.034) |
| Livestock unit | -0.004 | -0.004 | 0.009 | 0.006 | 0.000 |
|  | (0.009) | (0.008) | (0.006) | (0.006) | (0.008) |
| *Asset quintile (reference=poorest quintile)* |  |  |  |  |  |
| 2nd quintile | 0.395 | 0.249 | -0.050 | -0.010 | -0.067 |
|  | (0.243) | (0.161) | (0.146) | (0.122) | (0.201) |
| 3rd quintile | -0.043 | 0.294 | -0.238 | -0.136 | -0.081 |
|  | (0.222) | (0.185) | (0.180) | (0.150) | (0.222) |
| 4th quintile | -0.158 | -0.202 | -0.320* | -0.287* | 0.191 |
|  | (0.250) | (0.185) | (0.175) | (0.149) | (0.208) |
| Richest quintile | -0.250 | -0.229 | -0.267 | -0.256* | 0.278 |
|  | (0.228) | (0.261) | (0.173) | (0.154) | (0.218) |
| HH received transfer (=1) | -0.287 | -0.259* | -0.054 | -0.096 | -0.320** |
|  | (0.218) | (0.155) | (0.148) | (0.129) | (0.137) |
| HH received remittances (=1) | -0.050 | 0.107 | -0.201* | -0.133 | 0.096 |
|  | (0.182) | (0.118) | (0.109) | (0.082) | (0.132) |
| Land owned OR operated in last 12 months (acre) | -0.046** | 0.001 | -0.009 | -0.006 | 0.004 |
|  | (0.019) | (0.013) | (0.012) | (0.011) | (0.015) |
| HH has wage employment (=1) | -0.115 | -0.148 | 0.039 | -0.006 | 0.114 |
|  | (0.189) | (0.118) | (0.117) | (0.109) | (0.160) |
| No dwelling deprivation (=1) | -0.614*** | -0.310* | 0.086 | 0.008 | 0.372** |
|  | (0.207) | (0.161) | (0.115) | (0.097) | (0.158) |
| No cellphone (=1) |  |  |  | 1.148*** | 0.376 |
|  |  |  |  | (0.191) | (0.367) |
| Constant | -0.169 | -0.317 | -1.604 | -0.631 | 0.213 |
|  | (1.108) | (1.152) | (1.075) | (0.859) | (1.105) |
| **Observations** | **998** | **930** | **863** | **998** | **1064** |
| **Pseudo R-squared** | **0.272** | **0.075** | **0.053** | **0.081** | **0.079** |
| ***% of attrition per cause*** | ***7*** | ***7*** | ***25*** | ***39*** | ***6*** |

Source: IFPRI/MSR household surveys (January and June 2020). Note: ^/a^ Conditional on household participation. Standard errors in parentheses; ^/b^ Individual-level indicators including male, age, education and literacy level, major occupation, and major occupation pertain to the household head in the household-level attrition probits, and to the individual respondent in the individual-level attrition probit; significant at * p<0.10, ** p<0.05, *** p<0.01.

**Table A2. Definition and descriptive statistics (mean) of household- and individual-level indicators used**

1. Individual-level indicators

| **Indicator** | **Data** | **Period** | **BL** | **PS** |
| --- | --- | --- | --- | --- |
| Receipt of agriculture- or market-related information from various channels | binary | BL, PS | 0.42 | 0.63 |
| Receipt of health- or nutrition-related information from various channels | binary | BL, PS | 0.50 | 0.94 |
| Respect among household members ^/a^ | binary | BL, PS | 0.78 | 0.73 |
| Gender (male=1) | binary | BL | 0.43 |  |
| Marital status |  |  |  |  |
| Single | binary | BL | 0.06 |  |
| Married | binary | BL | 0.87 |  |
| Widow/widower/divorced | binary | BL | 0.07 |  |
| Education |  |  |  |  |
| No formal schooling | binary | BL | 0.30 |  |
| Primary school | binary | BL | 0.44 |  |
| High school or higher | binary | BL | 0.26 |  |
| Age | continuous | BL | 50.2 |  |
| Empowered ^/b^ | binary | BL | 0.58 |  |
| Empowerment score ^/b^ | continuous (0-1) | BL | 0.70 |  |
| Achieves work balance ^/c^ | binary | BL | 0.77 |  |
| Total hours spent a day on productive work (24-hour recall) | continuous (0-24) | BL | 7.21 |  |
| Hours spent of agriculture | continuous (0-24) | BL | 1.58 |  |
| Hours spent of nonfarm work | continuous (0-24) | BL | 1.08 |  |
| Hours spent on chore | continuous (0-24) | BL | 2.67 |  |
| Hours spent on child care | continuous (0-24) | BL | 0.47 |  |
| Hours spent on child care (while doing other work) | continuous (0-24) | BL | 0.34 |  |
| Hours spent on elderly care | continuous (0-24) | BL | 0.04 |  |
| Hours spent on commuting | continuous (0-24) | BL | 1.03 |  |
| Hours spent on leisure/sleep/rest | continuous (0-24) | BL | 17.13 |  |
| Change in stress or tension in the household during COVID than usual |  |  |  |  |
| Same | binary | PS (recall) |  | 0.73 |
| More | binary | PS (recall) |  | 0.23 |
| Less | binary | PS (recall) |  | 0.04 |
| Have no or rare disagreements within the household in the last 2 weeks (before survey), compared to having more frequent disagreements | binary | PS |  | 0.89 |
| Level of fear about effect of crisis on health | count (1-10) | PS |  | 6.96 |
| Low | binary | PS |  | 0.14 |
| Moderate | binary | PS |  | 0.37 |
| High | binary | PS |  | 0.49 |
| Level of pessimism about effect of crisis on income | count (1-10) | PS |  | 5.71 |
| Low | binary | PS |  | 0.24 |
| Moderate | binary | PS |  | 0.45 |
| High | binary | PS |  | 0.31 |
| Change in time spent on household chores and care during COVID |  |  |  |  |
| Decrease | binary | PS (recall) |  | 0.05 |
| Same | binary | PS (recall) |  | 0.56 |
| Increase | binary | PS (recall) |  | 0.39 |
| Change in time spent on crop farming during COVID |  |  |  |  |
| Decrease | binary | PS (recall) |  | 0.07 |
| Same | binary | PS (recall) |  | 0.80 |
| Increased | binary | PS (recall) |  | 0.13 |
| Change in time spent on wage and nonfarm work during COVID |  |  |  |  |
| Decrease | binary | PS (recall) |  | 0.27 |
| Same | binary | PS (recall) |  | 0.62 |
| Increase | binary | PS (recall) |  | 0.11 |
| Change in time spent across all household, farm, and nonfarm work |  |  |  |  |
| Decrease | binary | PS (recall) |  | 0.12 |
| Same | binary | PS (recall) |  | 0.50 |
| Increase | binary | PS (recall) |  | 0.38 |
| Farm wage work loss | binary | PS (recall) |  | 0.08 |
| Farm wage work gain | binary | PS (recall) |  | 0.02 |
| Farm wage work lost due to chores/childcare | binary | PS (recall) |  | 0.01 |
| Nonfarm wage work loss | binary | PS (recall) |  | 0.05 |
| Nonfarm wage work gain | binary | PS (recall) |  | 0.01 |
| N |  |  | 1069 | 1072 |

**(b) Household-level indicators**

| **Indicator** | **Data** | **Period** | **BL** | **PS** |
| --- | --- | --- | --- | --- |
| Woman-adult-only household (WHH) | binary | BL, PS | 0.15 | 0.10 |
| Household size | count | BL, PS | 4.07 | 4.67 |
| Number of children (age <18) | count | BL, PS | 1.03 | 1.09 |
| Receipt of remittance | binary | BL, PS | 0.32 | 0.25 |
| Remittance value (MM) | continuous | BL, PS | 994,813.37 | 184,446.52 |
| Receipt of government transfer | binary | BL, PS | 0.07 | 0.36 |
| Irrigation water user | binary | BL | 0.55 |  |
| Landed household | binary | BL, PS | 0.68 | 0.65 |
| No dwelling deprivation ^/d^ | binary | BL | 0.19 |  |
| Asset quintile 1 ^/e^ | binary | BL | 0.19 |  |
| Asset quintile 2 | binary | BL | 0.23 |  |
| Asset quintile 3 | binary | BL | 0.18 |  |
| Asset quintile 4 | binary | BL | 0.21 |  |
| Asset quintile 5 | binary | BL | 0.18 |  |
| HH has new adult / returning migrant | binary | BL | 0.37 |  |
| Gender parity within the household ^/f^ | binary | BL | 0.48 |  |
| NGO transfer | binary | PS |  | 0.08 |
| Transfer (government + NGO) | binary | PS |  | 0.39 |
| Income loss | binary | PS (recall) |  | 0.56 |
| Decreased quantity of meat consumption | binary | PS (recall) |  | 0.37 |
| Decreased quantity of fish consumption | binary | PS (recall) |  | 0.26 |
| Decreased quantity of meat and fish consumption | binary | PS (recall) |  | 0.41 |
| Decreased frequency of meat consumption | binary | PS (recall) |  | 0.39 |
| Decreased frequency of fish consumption | binary | PS (recall) |  | 0.29 |
| Decreased frequency of meat and fish consumption | binary | PS (recall) |  | 0.45 |
| **N** |  |  | **606** | **606** |

Source: IFPRI/MSR household surveys (January and June 2020). Note: BL=baseline survey; PS=phone survey; PS (recall) = no baseline collected, and respondents were asked to recall and compare indicators during COVID and before COVID or compared to usual or same time last year.

^/a^ One of the 12 indicators of the project-level or pro-WEAI methodology (Malapit et al. 2019) and a measure of intrahousehold harmony. It is defined as “How do you feel about some of other people in your household or family group and how do you think they feel about you?”, and a woman or a man is considered having achieved adequacy in this indicator if she or he meets ALL of the following conditions related to another household member: (1) Respondent respects relation (ALL or MOST of the time); (2) Relation respects respondent (ALL or MOST of the time); (3) Respondent trusts relation (ALL or MOST of the time); and (4) Respondent is comfortable disagreeing with relation (ALL or MOST of the time).

^/b^ For each of the 12 indicators of pro-WEAI, we estimate “adequacy” or whether a respondent satisfies a specific threshold, e.g. a person is adequate in the group membership indicator if she is a member of at least one group. A person is considered “empowered” if s/he is adequate in at least 9 out of 12 indicators.

^/c^ One of the 12 indicators of pro-WEAI. It is defined as “How many hours a day do you work? How much of that time is spent on care giving for children?” A woman or a man is considered having achieved adequacy in work balance if s/he works less than 10.5 hours per day. “Work” includes all livelihood activities and related travel, household chores, and care for children and elderly. All other activities, such as sleep, rest, relaxation, socialization, and sports, are considered as leisure and not “work”. See detailed in Annex Table A6.

^/d^ Based on a composite index for no deprivation on crowding (1-3 people per room), improved cooking fuels, and improved housing. UN-Habitat defines crowding, and therefore deprivation, when there are more than three people per room (UN 2007). Crowding indicator excludes kitchens, toilets, corridors, balconies, and rooms used only for business. Improved cooking fuels include electricity, liquified petroleum gas, or biogas. Improved housing is a house that is not a hut and does not have natural or rudimentary floors, walls, or roof. See Ragasa et al. (2020) for details.

^/e^ Asset scores are constructed through principal component analysis, and using various house furniture, vehicles, equipment, land, and livestock units owned by the household.

^/f^ Computed as empowerment score of male respondent minus empowerment score of female respondent (M –W) within the household. If women’s empowerment score is at least as equal to men’s, there is gender parity in the household.

|  |  |
| --- | --- |

**Table A3. Correlates of income loss, food consumption, and intrahousehold gaps in respect**

|  | (1) | (2) | | (3) | | | (4) | (5) | | | |  | | (6) | | | |  |  |
| --- | --- | --- | --- | --- | --- | --- | --- | --- | --- | --- | --- | --- | --- | --- | --- | --- | --- | --- | --- |
|  | Income loss (=1) | Reduced meat/fish quantity consumed  (=1) | | Reduced meat/fish consumption frequency (=1) | | | Task-sharing (=1) | Change in intrahousehold gap in respect (base=No change) | | | |  | | Intrahousehold gap in respect during PS (base: W=M) | | | |  |  |
|  |  |  | |  | | |  | Worsened (for any W/M) | | Improved (no decline for both) | |  | | (M>W) | (W>M) | | |  |  |
| WHH (=1) | 0.050 | 0.059 | | -0.047 | | |  |  | |  | |  | |  |  | | |  |  |
|  | (0.099) | (0.077) | | (0.096) | | |  |  | |  | |  | |  |  | | |  |  |
|  |  |  | |  | | |  |  | |  | |  | |  |  | | |  |  |
| Landed (=1) | -0.218*** | -0.055 | | -0.067 | | | -0.101 | -0.198 | | 0.489 | |  | | -0.766** | -0.300 | | |  |  |
|  | (0.072) | (0.068) | | (0.064) | | | (0.071) | (0.415) | | (0.365) | |  | | (0.347) | (0.473) | | |  |  |
|  |  |  | |  | | |  |  | |  | |  | |  |  | | |  |  |
| Nonpoor (not deprived n | 0.047 | -0.051 | | -0.073 | | | 0.015 | 0.014 | | -0.653** | |  | | -0.015 | 0.377 | | |  |  |
| dwelling) (=1) | (0.042) | (0.047) | | (0.058) | | | (0.047) | (0.224) | | (0.331) | |  | | (0.350) | (0.379) | | |  |  |
|  |  |  | |  | | |  |  | |  | |  | |  |  | | |  |  |
| Household size | 0.003 | 0.029** | | 0.028* | | | 0.024** | 0.046 | | 0.087 | |  | | -0.014 | -0.018 | | |  |  |
|  | (0.014) | (0.012) | | (0.015) | | | (0.011) | (0.073) | | (0.065) | |  | | (0.072) | (0.076) | | |  |  |
|  |  |  | |  | | |  |  | |  | |  | |  |  | | |  |  |
| HH has returning migrant (=1) | 0.198*** | -0.018 | | -0.008 | | | 0.046 | -0.038 | | -0.179 | |  | | -0.175 | -0.144 | | |  |  |
|  | (0.046) | (0.049) | | (0.040) | | | (0.046) | (0.255) | | (0.255) | |  | | (0.245) | (0.299) | | |  |  |
|  |  |  | |  | | |  |  | |  | |  | |  |  | | |  |  |
| HH has empowered woman (=1) | -0.044 | -0.026 | | 0.041 | | | 0.044 |  | |  | |  | |  |  | | |  |  |
|  | (0.052) | (0.045) | | (0.045) | | | (0.032) |  | |  | |  | |  |  | | |  |  |
|  |  |  | |  | | |  |  | |  | |  | |  |  | | |  |  |
| HH received transfer (=1) | 0.146** | -0.084 | | -0.012 | | | -0.028 | -0.100 | | 0.572* | |  | | 0.080 | -0.665 | | |  |  |
|  | (0.063) | (0.064) | | (0.057) | | | (0.055) | (0.413) | | (0.340) | |  | | (0.425) | (0.563) | | |  |  |
|  |  |  | |  | | |  |  | |  | |  | |  |  | | |  |  |
| HH experienced income loss (=1) |  | 0.245*** | | 0.278*** | | |  |  | |  | |  | |  |  | | |  |  |
|  |  | (0.048) | | (0.041) | | |  |  | |  | |  | |  |  | | |  |  |
|  |  |  | |  | | |  |  | |  | |  | |  |  | | |  |  |
| Irrigation site #2 (=1) | 0.052 | -0.063 | | 0.002 | | | -0.035 | 0.213 | | 0.526 | |  | | 0.514 | 0.071 | | |  |  |
|  | (0.054) | (0.054) | | (0.053) | | | (0.039) | (0.364) | | (0.370) | |  | | (0.361) | (0.322) | | |  |  |
|  |  |  | |  | | |  |  | |  | |  | |  |  | | |  |  |
| Constant |  |  | |  | | |  | -0.850 | | -2.039*** | |  | | -0.953 | -1.524** | | |  |  |
|  |  |  | |  | | |  | (0.592) | | (0.590) | |  | | (0.587) | (0.694) | | |  |  |
|  |  |  | |  | | |  |  | |  | |  | |  |  | | |  |  |
| *Observations* | | | *540* | | *540* | *540* | | | *426* | | *460* | |  | | |  | *460* | |  |
| *Pseudo R-squared* | | | *0.061* | | *0.058* | *0.069* | | | *0.022* | | *0.013* | |  | | |  | *0.021* | |  |

Source: IFPRI/MSR phone survey (June 2020). Note: Figures are marginal effects; standard errors clustered at village level in parentheses; models 1–4 are estimated using logit model and Models 5-6 are estimated using multinomial logit; significant at *** 1%, ** 5%, * 10% level.

**Table A4. Correlates of receipt of transfer**

|  | **(1)** | **(2)** | **(3)** | **(4)** |
| --- | --- | --- | --- | --- |
| **Explanatory variables** | **All** | **All** | **Site 1** | **Site 2** |
|  |  |  |  |  |
| WHH (=1) | 0.026 | 0.115* | 0.104* | -0.066 |
|  | (0.068) | (0.065) | (0.058) | (0.095) |
| Landed (=1) | -0.623*** |  | -0.499*** | -0.311*** |
|  | (0.085) |  | (0.108) | (0.105) |
| HH has new adult or returning migrant (=1) | 0.096** | 0.089** | 0.040 | 0.110** |
|  | (0.046) | (0.043) | (0.032) | (0.048) |
| Nonpoor HH (no dwelling deprivation) (=1) | -0.082** | -0.141*** | -0.024 | -0.118 |
|  | (0.039) | (0.038) | (0.022) | (0.082) |
| Number of kids (<18) | -0.018 | 0.004 |  |  |
|  | (0.015) | (0.018) |  |  |
| *Asset quintile (reference=poorest)* |  |  |  |  |
| Asset quintile 2 | -0.050 | -0.105 |  |  |
|  | (0.114) | (0.109) |  |  |
| Asset quintile 3 | 0.042 | -0.013 |  |  |
|  | (0.111) | (0.100) |  |  |
| Asset Quintile 4 | -0.004 | -0.062 |  |  |
|  | (0.103) | (0.097) |  |  |
| Asset quintile 5 (richest) | -0.001 | 0.018 |  |  |
|  | (0.047) | (0.054) |  |  |
| Irrigation site #2 (=1) | -0.639*** | -0.540*** |  |  |
|  | (0.095) | (0.104) |  |  |
|  |  |  |  |  |
| *Observations* | *606* | *606* | *372* | *234* |
| *Pseudo R-squared* | *0.411* | *0.297* | *0.375* | *0.058* |

Source: IFPRI/MSR phone survey (June 2020). Note: Figures are marginal effects; standard errors clustered at the village level in parentheses: models are estimated using logistic regression; significant at *** 1%, ** 5%, * 10% level.

**Table A5. Level of concern about COVID-19 (% of respondents)**

1. By gendered household type

|  | WHH | DHH | | | Gap |
| --- | --- | --- | --- | --- | --- |
|  | Women | Women | Men | Gender gap | Women (DHH vs. WHH) |
| Level of fear about the health effect of COVID-19 (not at all afraid=0, very afraid=10) | 6.9 | 7.4 | 6.9 | -0.5** | 0.4 |
| Level of pessimism about the effect of the crisis on your income (very optimistic=0, very pessimistic=10) | 5.0 | 5.4 | 5.6 | 0.2 | 0.4 |
| *N* | 69 | 508 | 495 |  |  |

1. By land ownership

|  | Landed households | | | Landless households | | | Gap | |
| --- | --- | --- | --- | --- | --- | --- | --- | --- |
|  | Women | Men | Gender gap | Women | Men | Gender gap | Women (Landed vs. Landless) | Men (Landed vs. Landless) |
| Level of fear about the health effect of COVID-19 (not at all afraid=0, very afraid=10) | 7.1 | 6.7 | -0.4* | 7.7 | 7.2 | -0.4 | -0.5 | -0.5 |
| Level of pessimism about the effect of the crisis on your income (very optimistic=0, very pessimistic=10) | 5.1 | 5.5 | 0.3 | 5.7 | 5.7 | 0.1 | -0.6 | -0.3 |
| *N* | 494 | 435 |  | 83 | 60 |  |  |  |

Source: IFPRI/MSR phone survey (June 2020). WHH=woman-adult-only household; DHH=dual-adult household; Statistically different at *** 1%, ** 5%, * 10% level of significance.

**Table A6. Correlates of individual level of stress, pessimism and fear of COVID effects**

|  | 1 | 2 | 3 | 4 |  | 5 |  | 6 |  | 7 |
| --- | --- | --- | --- | --- | --- | --- | --- | --- | --- | --- |
| Explanatory variables | Pessimism on economic impacts (1-10) | Fear of health impacts (1-10) | Respect (=1) | Discussions (base: the same) | | Input in discussions (base: the same) | | Stress (base: the same) | | Rare disagree-ments (=1) |
|  |  |  |  | Less than before | More than before | Less than before | More than before | More than usual | Less than usual |  |
| Male (=1) | 0.046* | -0.047* | 0.085*** | -0.126 | 0.019 | -0.069 | 0.140 | 0.269 | 0.657** | 0.017 |
|  | (0.028) | (0.025) | (0.027) | (0.392) | (0.146) | (0.385) | (0.153) | (0.179) | (0.294) | (0.020) |
| Age | -0.001 | -0.003*** | -0.002 | -0.011 | -0.028*** | -0.007 | -0.028*** | -0.021** | 0.003 | 0.001 |
|  | (0.001) | (0.001) | (0.001) | (0.018) | (0.007) | (0.018) | (0.007) | (0.008) | (0.014) | (0.001) |
| *Education (reference: at least high school)* |  |  |  |  |  |  |  |  |  |  |
| Did not complete primary (=1) | -0.079* | -0.003 | 0.003 | 2.066* | 0.031 | 14.182 | 0.135 | -0.533* | 0.743 | -0.023 |
|  | (0.046) | (0.042) | (0.046) | (1.073) | (0.244) | (477.287) | (0.259) | (0.286) | (0.597) | (0.035) |
| Primary school graduate (=1) | -0.011 | 0.035 | 0.019 | 1.281 | 0.047 | 13.834 | 0.235 | -0.178 | 0.784 | -0.011 |
|  | (0.043) | (0.038) | (0.043) | (1.066) | (0.222) | (477.287) | (0.234) | (0.252) | (0.571) | (0.032) |
| Received agriculture or market | -0.017 | 0.048* | 0.039 | 0.364 | 0.272* | 0.637 | 0.234 | 0.051 | -0.435 | -0.004 |
| information (=1) | (0.029) | (0.026) | (0.029) | (0.395) | (0.156) | (0.419) | (0.162) | (0.189) | (0.296) | (0.020) |
| WHH (=1) | 0.008 | -0.096 | 0.059 | 0.846 | -0.539 | 0.933 | 0.329 | 0.733* | 0.658 |  |
|  | (0.078) | (0.068) | (0.063) | (0.652) | (0.456) | (0.828) | (0.398) | (0.411) | (0.411) |  |
| Landed (=1) | -0.015 | -0.090** | 0.139*** | -0.854* | -0.305 | -0.318 | -0.146 | -0.209 | -0.358 | 0.048 |
|  | (0.046) | (0.040) | (0.052) | (0.503) | (0.241) | (0.577) | (0.247) | (0.278) | (0.455) | (0.038) |
| Nonpoor HH (no dwelling | -0.066** | 0.047 | 0.006 | 0.497 | -0.016 | -0.532 | -0.294 | -0.073 | -0.277 | 0.014 |
| deprivation) (=1) | (0.033) | (0.029) | (0.033) | (0.452) | (0.175) | (0.487) | (0.188) | (0.210) | (0.390) | (0.023) |
| HH had returning migrant (=1) | -0.033 | 0.012 | 0.003 | -0.559 | 0.151 | 0.219 | 0.128 | 0.066 | -0.206 | 0.005 |
|  | (0.030) | (0.026) | (0.029) | (0.439) | (0.158) | (0.396) | (0.164) | (0.190) | (0.332) | (0.021) |
| Household size | 0.032*** | 0.009 | -0.005 | -0.087 | 0.064 | 0.276*** | 0.040 | -0.038 | -0.106 | 0.009 |
|  | (0.008) | (0.007) | (0.008) | (0.121) | (0.044) | (0.105) | (0.047) | (0.054) | (0.094) | (0.006) |
| Empowered (=1) | -0.032 | -0.009 | -0.013 | 0.117 | 0.064 | -0.003 | 0.114 | 0.092 | 0.308 | 0.008 |
|  | (0.030) | (0.026) | (0.030) | (0.397) | (0.157) | (0.418) | (0.162) | (0.189) | (0.303) | (0.021) |
| Income loss (=1) | 0.207*** | 0.054** | 0.004 | 0.856** | 0.788*** | 0.916** | 0.599*** | 0.872*** | -0.408 | -0.049** |
|  | (0.028) | (0.025) | (0.028) | (0.408) | (0.151) | (0.418) | (0.157) | (0.188) | (0.309) | (0.020) |
| HH accepted from government | -0.065 | -0.040 | 0.063* | 0.411 | -0.189 | -0.382 | 0.001 | -0.014 | -0.191 | 0.032 |
| transfer (=1) | (0.041) | (0.034) | (0.037) | (0.521) | (0.214) | (0.606) | (0.217) | (0.266) | (0.394) | (0.025) |
| Household accepted an NGO/private | -0.143** | -0.007 | -0.080 | 1.225* | -0.032 | 0.402 | -0.371 | 0.175 | 0.637 | 0.021 |
| transfer (=1) | (0.063) | (0.049) | (0.063) | (0.662) | (0.306) | (0.821) | (0.323) | (0.367) | (0.502) | (0.036) |
| Irrigation site #2 (=1) | 0.140*** | -0.201*** | -0.018 | 0.894 | -0.004 | 0.410 | -0.170 | 0.525** | -0.212 | 0.039 |
|  | (0.037) | (0.031) | (0.035) | (0.544) | (0.192) | (0.572) | (0.197) | (0.245) | (0.357) | (0.027) |
| Constant | 1.473*** | 2.207*** |  | -4.517*** | -0.093 | -19.007 | -0.309 | -0.906 | -2.441** |  |
|  | (0.098) | (0.085) |  | (1.598) | (0.512) | (477.288) | (0.531) | (0.615) | (1.075) |  |
| *Observations* | *1025* | *1025* | *1021* | *1015* |  | *1015* |  | *1021* |  | *982* |
| *Pseudo R-squared* | *0.024* | *0.015* | *0.026* | *0.061* |  | *0.052* |  | *0.060* |  | *0.025* |

Source: IFPRI/MSR phone survey (June 2020). Note: Figures are marginal effects; standard errors clustered at the village level in parentheses; models 1-2 are estimated using Poisson regression; models 3,7 are estimated using logistic regression; and models 4-6 are estimated using multinomial logistic regression; significant at *** 1%, ** 5%, * 10% level.

**Table A7. Comparison of new DHH to other groups**

|  |  | All HH | | DHH in BL | | New DHH | Women in  DHH vs  New DHH | WHH | Women in  WHH vs  New DHH |
| --- | --- | --- | --- | --- | --- | --- | --- | --- | --- |
| Variable description | Reference period | W | M | W | M | W |  | W |  |
| Achieve work balance (%) | BL | 70.0 | 79.9 | 68.9 | 79.9 | 71.8 |  | 81.9 |  |
| Workload (hours) | BL | 8.0 | 6.6 | 8.1 | 6.6 | 7.4 |  | 6.8 |  |
| Time in own farm work (hours) | BL | 1.1 | 2.5 | 1.1 | 2.5 | 0.9 |  | 0.8 |  |
| Time in wage work / NFE (hours) | BL | 1.3 | 1.3 | 1.3 | 1.3 | 2.0 |  | 1.2 |  |
| Time in chores (hours) | BL | 3.5 | 1.1 | 3.6 | 1.1 | 2.8 | ** | 3.1 |  |
| Time in childcare (hours) | BL | 0.9 | 0.2 | 0.9 | 0.2 | 0.7 |  | 1.0 |  |
| Time in secondary childcare (1/2 hours) ^/a^ | BL | 0.6 | 0.2 | 0.6 | 0.2 | 0.8 |  | 0.4 |  |
| Time caring for adults (hours) | BL | 0.1 | 0.0 | 0.1 | 0.0 | 0.0 | *** | 0.0 |  |
| Time commuting (hours) | BL | 0.5 | 1.3 | 0.5 | 1.3 | 0.2 | ** | 0.3 |  |
| Time sleeping/resting/leisure (hours) | BL | 16.6 | 17.6 | 16.4 | 17.6 | 17.4 | * | 17.6 |  |
| Change in childcare hours (% of respondents) | Less than before | 6.5 | 4.4 | 5.6 | 4.4 | 4.0 |  | 10.1 |  |
|  | About the same as before | 62.0 | 61.9 | 62.0 | 61.9 | 38.5 |  | 78.5 | ** |
|  | More than before | 31.4 | 33.7 | 32.4 | 33.7 | 57.5 | * | 11.5 | *** |
| Change in elder care hours (% of respondents) | Less than before | 3.8 | 2.6 | 3.8 | 2.6 | 10.4 |  | 3.3 |  |
|  | About the same as before | 74.2 | 76.7 | 73.6 | 76.7 | 69.6 |  | 81.2 |  |
|  | More than before | 21.9 | 20.7 | 22.6 | 20.7 | 20.0 |  | 15.4 |  |
| Change in chores hours (% of respondents) | Less than before | 2.9 | 1.6 | 3.2 | 1.6 | 0.0 | *** | 1.3 |  |
|  | About the same as before | 70.7 | 79.4 | 70.1 | 79.4 | 58.0 |  | 79.8 |  |
|  | More than before | 26.4 | 19.0 | 26.7 | 19.0 | 42.0 |  | 18.9 |  |
| Change in own crop hours (% of respondents) | Less than before | 7.3 | 5.6 | 7.9 | 5.6 | 0.0 | *** | 3.3 |  |
|  | About the same as before | 87.3 | 76.9 | 87.5 | 76.9 | 90.0 |  | 81.0 |  |
|  | More than before | 5.4 | 17.6 | 4.5 | 17.6 | 10.0 |  | 15.8 |  |
| Change in wage work / NFE hours (% of respondents) | Less than before | 19.2 | 27.9 | 17.0 | 27.9 | 24.7 |  | 27.8 |  |
|  | About the same as before | 70.7 | 66.4 | 72.5 | 66.4 | 48.9 |  | 70.1 |  |
|  | More than before | 10.2 | 5.6 | 10.6 | 5.6 | 26.3 |  | 2.0 | ** |
| Net change in chores and care hours (% of respondents) | Less than before | 7.1 | 3.8 | 7.0 | 3.8 | 3.1 |  | 6.2 |  |
|  | About the same as before | 54.1 | 60.8 | 52.8 | 60.8 | 50.7 |  | 67.8 |  |
|  | More than before | 38.8 | 35.4 | 40.2 | 35.4 | 46.3 |  | 26.0 |  |
| Net change in household and farm work hours (% of respondents) | Less than before | 9.2 | 5.3 | 9.4 | 5.3 | 3.1 |  | 6.2 |  |
|  | About the same as before | 52.5 | 55.4 | 51.0 | 55.4 | 50.7 |  | 67.9 |  |
|  | More than before | 38.4 | 39.3 | 39.6 | 39.3 | 46.3 |  | 26.0 |  |
| Net change in all work (% of respondents) | Less than before | 11.5 | 10.5 | 11.8 | 10.5 | 3.1 | ** | 8.0 |  |
|  | About the same as before | 50.9 | 51.2 | 48.9 | 51.2 | 46.9 |  | 71.7 | * |
|  | More than before | 37.6 | 38.3 | 39.2 | 38.3 | 50.0 |  | 20.3 | * |
| Farm wage work loss (%) | change | 9.1 | 7.7 | 8.1 | 7.7 | 15.7 |  | 15.3 |  |
| Farm wage work gain (%) | change | 2.7 | 3.2 | 2.9 | 3.2 | 3.2 |  | 1.0 |  |
| Farm wage work lost due to chores/childcare (%) | change | 1.4 | 0.2 | 0.6 | 0.2 | 10.3 |  | 4.6 |  |
| Nonfarm wage work loss (%) | change | 4.2 | 5.0 | 3.8 | 5.0 | 4.9 |  | 7.6 |  |
| Nonfarm wage work gain (%) | change | 0.4 | 1.6 | 0.5 | 1.6 | 0.0 | ** | 0.0 |  |
| Level of fear about effect of crisis on health (1-10) | PS | 7.3 | 6.9 | 7.4 | 6.9 | 7.7 |  | 6.7 |  |
| Level of pessimism about effect of crisis on income (1-10) | PS | 5.3 | 5.6 | 5.4 | 5.6 | 4.1 |  | 5.3 |  |
| Achieve respect in relationships (%) | BL | 72.0 | 77.7 | 72.1 | 77.6 | 76.6 |  | 59.6 |  |
|  | PS | 68.7 | 76.7 | 67.3 | 76.7 | 79.0 |  | 84.1 |  |
| Change in adequacy in respect: PS>BL | change | 15.6 | 11.7 | 14.3 | 11.7 | 7.5 |  | 37.2 | *** |
| Change in adequacy in respect: PS<BL | change | 20.4 | 15.0 | 21.3 | 15.0 | 21.0 |  | 7.3 |  |
| Change in adequacy in respect: PS=BL | change | 64.1 | 73.3 | 64.3 | 73.3 | 71.5 |  | 55.5 |  |
| Change in household tension (%) | Less than before | 4.4 | 6.5 | 4.8 | 6.5 | 0.0 | *** | 0.0 |  |
|  | About the same as before | 78.6 | 75.2 | 78.9 | 75.2 | 15.5 | *** | 84.9 | *** |
|  | More than before | 17.0 | 18.3 | 16.3 | 18.3 | 84.5 | *** | 15.1 | *** |
| Fight with partner: Rarely (%) | PS | 86.2 | 89.6 | 85.1 | 89.6 | 100.0 | *** | 100.0 |  |
| Fight with partner: Sometimes (%) | PS | 12.7 | 9.6 | 13.6 | 9.6 | 0.0 | *** | 0.0 |  |
| Fight with partner: Often (%) | PS | 1.1 | 0.8 | 1.2 | 0.8 | 0.0 | ** | 0.0 |  |
| Number of individuals | BL | 998 | 855 | 880 | 854 | 72 |  | 45 |  |
| Number of individuals responding to intra-hh harmony questions | BL | 939 | 855 | 880 | 854 | 30 |  | 28 |  |
| Number of individuals | PS | 577 | 495 | 507 | 495 | 24 |  | 45 |  |
| Number of individuals responding to intrahousehold harmony questions | PS | 544 | 489 | 506 | 489 | 9 |  | 28 |  |

Source: IFPRI/MSR household surveys (January and June 2020). Note: The figures are averages, and are significantly different between groups at *** 1%, ** 5%, * 10% level; BL=baseline survey; PS=phone survey; ^/a^ The survey questionnaire on time use asked about child care as primary work and child care while doing other work. Following the WEAI methodology, we used 0.5 as weight to apportion the hour spent on child care while doing other work and added to the hours of child care as primary work. See also Annex Table A7.

**Table A8. Reported hours spent on various activities (24-hour recall)**

|  | All households | | | Landed households | | | Landless households | | | Gap | |
| --- | --- | --- | --- | --- | --- | --- | --- | --- | --- | --- | --- |
| Activity | Women | Men | Gender gap | Women | Men | Gender gap | Women | Men | Gender gap | Women (Landed vs Landless) | Men (Landed vs Landless) |
| Agricultural work (hrs) | 1.1 | 2.5 | 1.5*** | 1.4 | 3.2 | 1.9*** | 0.5 | 0.8 | 0.3 | 0.9*** | 2.4*** |
| Nonagricultural work (hrs) | 1.3 | 1.3 | -0.0 | 0.8 | 0.8 | -0.1 | 2.3 | 2.7 | 0.4 | -1.5*** | -2.0*** |
| Commuting (hrs) | 0.5 | 1.3 | 0.8*** | 0.6 | 1.3 | 0.7*** | 0.4 | 1.2 | 0.8* | 0.2 | 0.1 |
| Household chores (hrs) | 3.5 | 1.1 | -2.4*** | 3.4 | 0.9 | -2.5*** | 3.6 | 1.5 | -2.2*** | -0.2 | -0.5 |
| Childcare as a primary activity (hrs) | 0.9 | 0.2 | -0.7*** | 0.8 | 0.2 | -0.6*** | 1.2 | 0.2 | -1.0*** | -0.3 | -0.0 |
| Childcare as a secondary activity (hrs) ^/a^ | 0.6 | 0.2 | -0.4*** | 0.5 | 0.2 | -0.3*** | 0.8 | 0.3 | -0.5** | -0.3 | -0.1 |
| Elder care (hrs) | 0.1 | 0.0 | -0.0* | 0.1 | 0.0 | -0.1* | 0.0 | 0.0 | -0.0 | 0.1*** | 0.0 |
| Sleeping/eating/leisure | 16.6 | 17.6 | 1.0*** | 16.9 | 17.6 | 0.7** | 16.0 | 17.6 | 1.6** | 0.9* | -0.1 |
| Total workload | 8.0 | 6.6 | -1.4*** | 7.6 | 6.6 | -1.0*** | 8.7 | 6.6 | -2.1*** | -1.1** | -0.0 |
| Achieving adequacy in work balance (%) ^/b^ | 70.0 | 79.9 | 10.0*** | 73.0 | 80.8 | 7.8** | 64.1 | 77.8 | 13.7** | 8.8* | 3.0 |
| *N* | 998 | 855 |  | 856 | 758 |  | 142 | 97 |  |  |  |

Source: IFPRI/MSR household survey (January 2020). Note: Significant at *** 1%, ** 5%, * 10% level. ^/a^ The survey questionnaire on time use asked about child care as primary work and child care while doing other work. Following the WEAI methodology, we used 0.5 as weight to apportion the hour spent on child care while doing other work and added to the hours of child care as primary work. ^/b^ This indicator is one of the 12 indicators of pro-WEAI. It is defined as “How many hours a day do you work? How much of that time is spent on care giving for children?” A woman or a man is considered having achieved adequacy in work balance if s/he works less than 10.5 hours per day. “Work” includes all livelihood activities and related travel, household chores, and care for children and elderly. All other activities, such as sleep, rest, relaxation, socialization, and sports, are considered as leisure and not “work.”

**Table A9. Change in time spent in household and employment activities and change in inputs to decision-making within household (% of households)**

|  | Gender relation | Chores | Childcare | Elderly care | Net chores and care | Farm work | Wage/ nonfarm enterprise work | Net household and farm work | Net household, farm, wage, and enterprise work | Inputs in decisions |
| --- | --- | --- | --- | --- | --- | --- | --- | --- | --- | --- |
| 1 | Both W and M increased | 7.6 | 17.5 | 8.6 | 21.2 | 2.3 | 1.5 | 21.5 | 20.3 | 12.5 |
| 2 | W increased, M had the same | 17.7 | 14.6 | 12.1 | 16.5 | 2.2 | 2.9 | 14.7 | 12.5 | 13.2 |
| 3 | W increased, M decreased | 0.6 | 0.3 | 0.2 | 1.2 | 0.5 | 7.0 | 2.3 | 5.5 | 0.3 |
| 4 | W had the same, M increased | 11.8 | 16.5 | 12.5 | 13.8 | 13.3 | 3.7 | 16.2 | 15.6 | 12.6 |
| 5 | Both W and M had the same | 57.8 | 44.6 | 60.4 | 39.0 | 68.1 | 48.9 | 34.1 | 30.9 | 56.9 |
| 6 | W had the same, M decreased | 1.1 | 1.6 | 2.2 | 1.6 | 5.3 | 18.2 | 2.1 | 3.5 | 1.8 |
| 7 | W decreased, M increased | 0.1 | 1.0 | 0.2 | 1.5 | 1.9 | 0.6 | 2.6 | 3.2 | 1.1 |
| 8 | W decreased, M had the same | 3.2 | 1.4 | 3.6 | 4.2 | 6.2 | 12.4 | 5.3 | 6.6 | 1.2 |
| 9 | Both W and M decreased | 0.0 | 2.4 | 0.2 | 1.1 | 0.2 | 4.7 | 1.2 | 2.0 | 0.3 |
|  | W increased (1–3) | 25.9 | 32.5 | 20.9 | 38.9 | 5.0 | 11.4 | 38.5 | 38.2 | 26.0 |
|  | M increased (1,4,7) | 19.5 | 35.1 | 21.2 | 36.5 | 17.6 | 5.8 | 40.3 | 39.0 | 26.3 |
|  | Household task-sharing (4,7) | 11.9 | 17.5 | 12.6 | 15.3 | 15.2 | 4.3 | 18.8 | 18.8 | 13.7 |
|  | W decreased (7–9) | 3.4 | 4.8 | 4.0 | 6.7 | 8.2 | 17.7 | 9.1 | 11.8 | 2.6 |
|  | N | 466 | 322 | 312 | 466 | 407 | 203 | 466 | 466 | 461 |

Source: IFPRI/MSR phone survey (June 2020). W=women; M=men; Statistically different at *** 1%, ** 5%, * 10% level of significance.

**Table A10. Correlates of time use change during COVID**

|  | Increased time spent on household work (base=no change) | | |  | Decreased time spent on household work (base=no change) | | |
| --- | --- | --- | --- | --- | --- | --- | --- |
|  | Marginal effects | Std. Err. |  |  | Marginal effects | Std. Err. |  |
| Workload (hours) | 0.008 | 0.004 | * |  | 0.001 | 0.002 |  |
| Male (=1) | -0.044 | 0.030 |  |  | -0.025 | 0.017 |  |
| Age | -0.006 | 0.002 | *** |  | 0.001 | 0.001 |  |
| No formal schooling (=1) ^/a^ | -0.006 | 0.054 |  |  | -0.019 | 0.027 |  |
| Primary school (=1) ^/b^ | -0.014 | 0.053 |  |  | -0.002 | 0.025 |  |
| Single (=1) ^/b^ | -0.069 | 0.069 |  |  | 0.010 | 0.026 |  |
| Married (=1) ^/b^ | -0.078 | 0.081 |  |  | -0.016 | 0.030 |  |
| Empowered (=1) | 0.016 | 0.033 |  |  | 0.049 | 0.022 | ** |
| WHH (=1) | -0.176 | 0.104 | * |  | -0.025 | 0.051 |  |
| Landed (=1) | -0.005 | 0.041 |  |  | -0.018 | 0.019 |  |
| No dwelling deprivation (=1) | 0.109 | 0.050 | ** |  | 0.004 | 0.015 |  |
| With new adult / returning migrant (=1) | 0.037 | 0.034 |  |  | 0.027 | 0.019 |  |
| Household size | 0.011 | 0.007 | * |  | 0.001 | 0.004 |  |
| Government transfer (=1) | -0.015 | 0.055 |  |  | 0.018 | 0.018 |  |
| NGO transfer (=1) | 0.037 | 0.046 |  |  | -0.090 | 0.044 | ** |
| Irrigation site #2 (=1) | 0.023 | 0.035 |  |  | -0.006 | 0.019 |  |
| N | 1208 |  |  |  |  |  |  |
| Pseudo R2 | 0.047 |  |  |  |  |  |  |

Source: IFPRI/MSR household surveys (January and June 2020). Note: Estimated using multinomial logit model; significant at *** 1%, ** 5%, * 10% level. ^/a^ base=at least high school; ^/b^ base=married.
